# Engineering *Saccharomyces boulardii* for enhanced surface display capacity

**DOI:** 10.1186/s12934-025-02702-3

**Published:** 2025-04-01

**Authors:** Luping Xu, Xingjian Bai, Deokyeol Jeong, Dahye Lee, Fransheska Semidey, Chenhai Li, Eun Joong Oh

**Affiliations:** 1 Department of Food Science, Purdue University, West Lafayette, IN 47907 USA; 2 Whistler Center for Carbohydrate Research, Purdue University, West Lafayette, IN 47907 USA; 3 VectorBuilder Inc., Chicago, IL 60609 USA

## Abstract

*Saccharomyces boulardii* (*Sb*) has gained significant attention for its potential therapeutic application as a probiotic yeast strain. Current approaches often leverage its secretion and display capabilities to deliver therapeutic agents aimed at alleviating intestinal disorders. However, relatively few studies have focused on optimizing its display efficiency. In this study, we evaluated two surface display systems, Aga2- and Sed1-based, for use in *Sb* by systematically modifying display cassette components and the host strain. Initially, both systems were tested in *Saccharomyces cerevisiae* (*Sc*) and *Sb* to validate their design. *Sc* consistently outperformed *Sb* in both display expression and efficiency, highlighting the need for further optimization in *Sb*. To enhance the display efficiency in *Sb*, we investigated specific modifications to the display cassette, including the use of linker sequences for Aga2 and variations in anchor length for Sed1. These experiments identified key factors influencing display performance. Subsequently, we engineered a modified *Sb* strain, LIP02, by overexpressing *AGA1* and deleting cell wall-related genes (*CCW12*, *CCW14*, and *FYV5*). These modifications were expected to expand the available docking sites for the protein of interest (POI) and improve overall protein secretion and display efficiency. As a result, the modified strain exhibited a significant enhancement in display capacity compared to the wild-type *Sb* strain. Furthermore, genome integration of the display cassette in LIP02 enhanced both stability and expression compared to plasmid-based systems. Importantly, the functionality of β-glucosidase displayed on LIP02 was preserved, as demonstrated by improved enzymatic activity and robust growth on cellobiose as the sole carbon source. These findings establish LIP02 as a superior host for surface display applications in *Sb*, offering a more stable and efficient platform for the expression of therapeutic proteins and other functional biomolecules.

**Supplementary Information:**

The online version contains supplementary material available at 10.1186/s12934-025-02702-3.

## Background

Yeast display is a versatile platform in biotechnology, enabling the immobilization of heterologous proteins on the yeast surface for rapid assessment of protein specificity and ligand affinity [1]. The spatial proximity of displayed proteins may also enhance their activity through synergistic effects [2]. This technology has been widely applied in diverse fields, including antibody engineering [3–5], biofuel production [6], bioremediation [7], and the development of whole-cell vaccines [8, 9]. Traditionally, *Saccharomyces cerevisiae* (*Sc*) has been the preferred host for yeast display due to its Generally Recognized as Safe (GRAS) status and the availability of extensive genome engineering tools. However, for applications targeting human health, *Saccharomyces boulardii* (*Sb*), a well-established probiotic with GRAS status, offers unique advantages. These include protection against pathogen colonization [10], immunomodulatory effects [11], and modulation of the commensal microbiome [12, 13], making it a promising alternative host for yeast display systems.

Recent advancements in bioengineered *Sb* have demonstrated its potential as a therapeutic agent, leveraging its probiotic and prebiotic properties [14–17]. Most applications have focused on in vivo therapies. For instance, Chen et al. engineered *Sb* to secrete neutralizing antibodies against *Clostridioides difficile* toxins. When orally administered to mice, the engineered strain exhibited both prophylactic and therapeutic effects, improving survival rates, reducing gastrointestinal inflammation, and decreasing *C. difficile* shedding [17]. Similarly, Durmusoglu et al. developed a bioengineered *Sb* strain capable of producing violacein and β-carotene in vivo. Despite these advancements, studies specifically exploring *Sb* as a platform for yeast display remain limited.

Due to the genomic similarity between *Sc* and *Sb* [18, 19], yeast display systems designed for *Sc* are transferable to *Sb*. The most commonly employed configuration for *Sc* display is the Aga1-Aga2 system, derived from the subunits of the cell surface glycoprotein heterodimer, a-agglutinin. In this system, the protein of interest (POI) is fused with Aga2, which forms disulfide bonds with Aga1, anchoring the POI to the cell surface. To date, several studies have investigated *Sb* display systems using the Aga1-Aga2 configuration [20, 21]. In one study, a co-expressing vector for Aga1 and Aga2 was constructed to demonstrate two key findings: first, the minor genomic differences between *Sc-* and *Sb*-derived Aga1 did not affect display functionality; second, the system successfully displayed enhanced green fluorescent protein (EGFP) and EtMic2 (chicken *Eimeria tenella *Microneme-2 protein), as confirmed by microscopy, flow cytometry, and Western blot analysis. However, this study did not assess the functional activity of the displayed proteins [21]. Another study employed a similar vector to display pancreatic lipase inhibitor peptides on *Sb*. The engineered strain significantly reduced pancreatic lipase activity in vitro and conferred weight loss and lower serum lipid levels in obese mice [20].

Despite its utility, the Aga1-Aga2 system has limitations, particularly the instability of disulfide bonds in reducing environments such as the human gut or fermentation processes [22]. Disulfide bonds between cysteine residues play a key role in stabilizing protein structures. The reduction and oxidation of these bonds induce conformational changes that enable proteins to switch between distinct functional states [23]. In the human gut, particularly in the large intestine (colon), a reducing environment prevails due to the dense microbial population that consumes oxygen and generates reducing agents [24]. Under such conditions, disulfide bonds in the Aga1-Aga2 system may undergo reduction, leading to protein destabilization and altered function of the displayed protein. This structural instability could compromise the efficacy of probiotic strains engineered for gut health. Since *Sb* exerts its effects through probiotic and prebiotic properties, the Aga1-Aga2 system alone may not provide a stable and functional platform for developing robust probiotic strains. These challenges have driven the search for alternative anchoring systems for yeast display. Several alternative anchor proteins have been explored for yeast display in *Sc*. Commonly used anchors include Sag1p, involved in mating-related cell aggregation [22]; Sed1p, which stabilizes the cell wall structure [25, 26]; Flo1p, a mediator of cell-cell interaction during flocculation [22, 27, 28]; and Cwp1p and Cwp2p, components of the cell wall [29]. To date, only one study has investigated alternative anchor proteins for *Sb* display. Of the six anchor proteins tested, Sed1p demonstrated superior display efficiency in *Sb* [30]. 

The lack of an optimized *Sb* display system limits its potential as a host for functional protein display. Given the advantages of bioengineered *Sb* as a probiotic, the development of a well-characterized *Sb* display platform is essential. Display optimization can be achieved through two approaches: (1) engineering the display cassette by modifying key elements such as the signal peptide [31–33], linker [22, 32, 34], or anchor protein length [27]; and (2) engineering the host strain to enhance protein secretion and display capacity. In this study, we report the development of an optimized *Sb* strain, LIP02, which features *AGA1* overexpression and deletions of *CCW12*, *CCW14*, and *FYV5*. LIP02 exhibited a three-fold increase in display efficiency, as indicated by Alexa 594 mean fluorescence intensity (MFI) measured by flow cytometry, compared to its parental strain, *Sb* ATCC MYA 797 *ura3Δ*. Using β-glucosidase as a model protein, we demonstrated that LIP02 exhibited a two-fold higher specific cellobiose consumption rate at 24 h. These findings highlight that our modifications significantly enhanced *Sb*’s display capacity without compromising the functionality of the displayed protein.

## Materials and methods

### Strains and media

*S. boulardii* ATCC MYA 797 and *S. cerevisiae* BY4741 (*MATa his3Δ1 leu2Δ0 met15Δ0 ura3Δ0*) strains were used as host strains to evaluate display efficiency. Both strains were routinely grown in yeast extract-peptone (YP) medium (10 g/L yeast extract, 20 g/L peptone) containing 20 g/L of glucose (YPD) at 30 °C. *Escherichia coli* Top10 was employed for plasmid propagation and grown in Luria-Bertani medium (LB) at 37 °C. Additional media were prepared as follows: YPD-N-H, which consisted of YPD supplemented with 300 µg/mL hygromycin B and 100 µg/mL nourseothricin; YPD-N, which included YPD with 100 µg/mL nourseothricin; YPC (20 g/L) and YPC (40 g/L), representing YP medium with 20 g/L and 40 g/L cellobiose, respectively; LBA, which was LB supplemented with 100 µg/mL ampicillin; and SC-ura, a synthetic complete medium (SC) lacking uracil.

### Plasmid construction and transformation

**Fig. 1:**
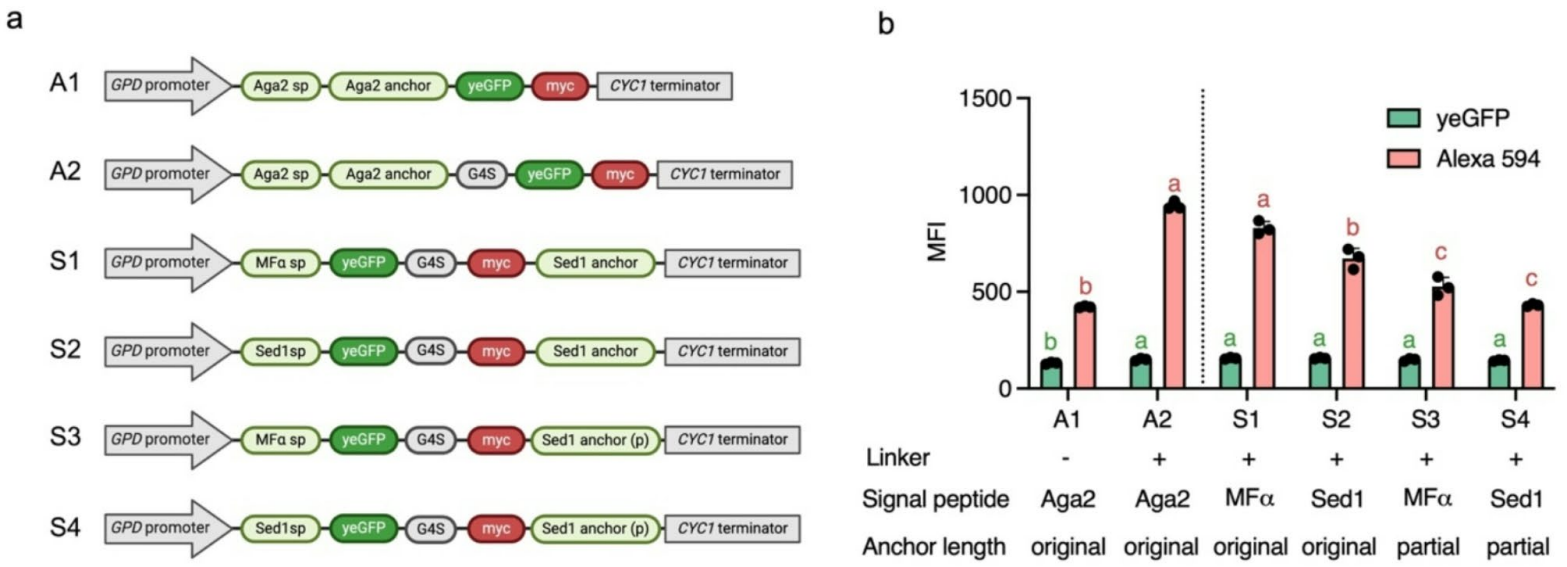
Evaluation of Aga2- and Sed1-based display systems in *Saccharomyces cerevisiae* (*Sc*) and *Saccharomyces boulardii* (*Sb*). (**a**) Schematic representation of the display cassettes expressed by plasmids. Each construct includes a signal peptide (Aga2sp, MFαsp, or Sed1sp), a G4S linker, yeGFP as a reporter protein, a myc tag for detection, and an anchor protein (Aga2 anchor or Sed1 anchor). Expression is driven by the *GPD* promoter with termination regulated by the *CYC1* terminator. (**b**) Flow cytometry analysis of *Sb* expressing Aga2- or Sed1-based plasmids. Mean fluorescence intensity (MFI) values for yeGFP (green) and Alexa 594 (red) were analyzed separately. Each analysis was conducted with 10,000 cells. Error bars represent standard deviation (SD), and statistical significance was determined using one-way ANOVA (*p* < 0.05). Different lowercase letters denote significant differences, with green letters corresponding to yeGFP MFI and red letters corresponding to Alexa 594 MFI

A customized plasmid, VB221020-1396kuv (Table S1), containing a *URA3* selection marker, 2µ origin, *GPD* (*TDH3*) promoter, and *CYC1* terminator, was constructed by Vector Builder Inc. and served as the backbone for constructing plasmids expressing the display cassette shown in Fig. 1a. The NEBridge^®^ Golden Gate Assembly Kit (BsmBI-v2) was used to integrate various display components into the backbone, flanked by the *GPD* promoter and *CYC1* terminator. All fragments were amplified using polymerase chain reaction (PCR) from specific sources: signal peptides, the G4S linker, and *AGA2* fused with an HA tag from pCTcon2 (Addgene #41843); *SbSED1* from *S. boulardii* ATCC MYA 797; and yeast-enhanced green fluorescent protein (yeGFP) from the pCYC1m_yeGFP (Addgene #64389). Myc-tags were added to the respective gene fragments through PCR. The design of the display cassette is shown in Fig. 1a, with the open reading frame (ORF) sequences provided in Table S1.

For the construction of Aga2-based *aaBGL* plasmid, β-glucosidase from *Aspergillus aculeatus* (*aaBGL*) was amplified from pPAP_BGL1(Addgene #183735) and integrated into a customized plasmid backbone (VB231023-1165htk) (Table S1) using the NEBridge^®^ Golden Gate Assembly Kit (BsmBI-v2). The design of the *aaBGL* display cassette is shown in Fig. 4a, and the ORF sequence is listed in Table S1. All plasmids were verified through whole-plasmid sequencing provided by Eurofins. All plasmids, containing a *URA3* selective marker, were transformed into the *S. boulardii* ATCC MYA 797 *ura3Δ* strain, which was generated via CRISPR/Cas9-mediated knockout as described in the subsequent section. Yeast transformations were performed using the lithium acetate method [35].

### Host modification and genome integration using CRISPR/Cas9

The plasmid pRS42H [36], harboring a hygromycin resistance marker, was used to deliver spacers and the gRNA scaffold. Spacers were designed via ChopChop (https://chopchop.cbu.uib.no/) and synthesized with flanking homologous arms for assembly into pRS42H using the NEBuilder HiFi assembly master mix (E2621L). Spacer sequences with homologous arms are detailed in Table S2. Donor DNA flanked by homologous regions was delivered as dsDNA fragments, with sequences also listed in Table S2. Oligonucleotide synthesis was performed by Azenta. The strains *Sb ura3Δ*, *ccw12Δ*, and *ccw14Δ* were constructed by co-transforming pCas9-NAT [37], pRS42H [36] carrying the respective 20 bp spacer, and matching donor DNA into *Sb* MYA 797. For *AGA1* overexpression, the native *AGA1* promoter was replaced with the *GPD* promoter, amplified from the plasmid VB221020-1396 (Table S1) using primers listed in Table S2. *Sb fyv5Δ* was constructed by introducing premature stop codons (TAGTGATAG) with a two-nucleotide frameshift, as clean knockout was unsuccessful. All engineered strains were confirmed by PCR using primers detailed in Table S2.

All genome-integration display cassettes were integrated into the intergenic site *int#11* (Chromosome XV 672412–674351), located between *DCI1* and *SYC1* on chromosome XV. The ORFs from plasmids expressing A2 and *aaBGL* (Table S1), along with the *GPD* promoter and *CYC1* terminator, were amplified using primers genome integration_donor_F and genome integration_donor_R (Table S3) to generate donor DNA. The plasmid pRS42H, containing a 20 bp gRNA spacer (TGTGACAAAATAGAATCCAG) targeting *int#11*, was co-transformed with the donor DNA into *Sb* MYA 797 *ura3Δ* and LIP02 strains harboring pCas9-NAT to construct genome-integrated *Sb* strains. The CRISPR-based transformation efficiency for *Sb* in this study typically ranges from 3.3 × 10⁴ to 6.7 × 10⁵ CFU/µg donor DNA, using the gRNA and donor DNA described in this section.

### Assessment of expression level and display efficiency

Frozen yeast transformants were streaked onto SC-ura plate and incubated at 30 °C for 48 h. Three single colonies were picked and inoculated into SC-ura medium as biological replicates. Cultures were grown at 30 °C, 250 rpm, for 48 h until reaching an optical density at 600 nm (OD_600_) of 4. Cells (200 µL) were collected by centrifugation, washed with sterilized water, and incubated with anti-myc antibody (Invitrogen MA1-980, 1:100, prepared in 5% BSA in PBS containing 0.05% Tween 20) at 30 °C for 2 h with constant shaking. After washing with sterilized water, cells were incubated with Alexa Fluor^®^ 594-conjugated anti-rabbit antibody (Abcam 150080, 1:200, prepared in 5% BSA in PBS containing 0.05% Tween 20) for 1.5 h at 30 °C with constant shaking, then washed and resuspended in sterilized water. Alternatively, for the genome-integrated strain, an anti-HA antibody conjugated with Alexa Fluor^®^ 555 (Thermo Fisher #26183-A555) was used for single-antibody labeling. This alternative labeling procedure reduced the total labeling time from 3.5 h to 1.5 h and further confirmed that the display efficiency was independent of the antibody choice or protein tag used. Display efficiency and expression levels were analyzed using a BD Fortessa LSR flow cytometry (Purdue Bindley Bioscience Center). Display efficiency was measured by Alexa 594 intensity (excitation 561 nm, detection 610/20, 585LP) or Alexa 555 intensity (excitation 561 nm, detection 586/15), while expression level was evaluated based on yeGFP intensity (excitation 488 nm, detection 530/30, 505LP). Flow cytometry gating was established using wild-type *Sb* (*Sb* WT) labeled with antibodies but lacking an inserted expression cassette. The gating threshold was set so that 99% of *Sb* WT cells fell within the negative population, while 1% were classified as positive. Fluorescence signals within the positive gate were considered indicative of target protein display. In each experiment, forward scatter (FSC) and side scatter (SSC) voltage settings were adjusted to ensure all events remained within plot boundaries, enabling optimal visualization of the cell population.

### Strain stability test

LIP02 carrying the plasmid expressing A2 cassette was cultured in YPD medium, and the strain underwent five serial transfers in YPD medium without selective pressure. The culture was then streaked onto YPD agar to obtain single colonies. Sixteen single colonies were randomly selected and streaked on both YPD and SC-ura agar plates. After overnight incubation at 30 °C, images of both plates were captured. To evaluate the stability of genome-integrated strains, LIP02, with the A2 cassette integrated into the genome, was recovered in YPD and underwent five serial transfers in YPD. The first and fifth subcultures were streaked onto YPD agar, and colony PCR was conducted using genome integration_donor primers listed in Table S3 to verify the genome-integrated expression cassette.

### Microscopic imaging

Fluorescence microscopy was employed as a quality control step prior to each experiment to verify plasmid expression in the strains. A single colony was mixed with 1 µL of water on microscope slides and observed using a Leica fluorescence microscope at 400× magnification. Confocal microscopy was used to evaluate the display levels, following the Alexa 594 antibody labeling protocol as flow cytometry sample preparation. Images were acquired with an A1Rsi Nikon confocal microscope equipped with 488 nm/561nm lasers and processed using Nikon Elements software (Nikon Instruments) at the Purdue Bindley Bioscience Imaging Facility.

### Cellobiose degradation assay

The wild-type (WT) and LIP02 strains harboring the *aaBGL* display cassette integrated at *int#11* were streaked on agar plates. A single colony was inoculated into YP medium containing 20 g/L of cellobiose (YPC) and incubated for 48 h. Cultures were normalized to an OD_600_ of 1 in YPC medium to a total volume of 20 mL and further incubated at 30 °C for 72 h at 250 rpm. Samples were collected at 0, 12, 18, 24, 32, 36, and 48 h for cellobiose quantification using high-performance liquid chromatography (HPLC, Agilent Technologies 1260 Infinity II) with a Rezex ROA-Organic Acid H^+^ (8%) column (Phenomenex Inc., Torrance, CA). The column was eluted with 0.005 N H_2_SO_4_ at 50 °C, and a flow rate was set at 0.5 mL/min. Three independent experiments were conducted, with samples collected at each time point.

### Specific cellobiose consumption rate assessment

*Sb* MYA 797 and LIP02 were cultivated in YPD medium. Upon reaching confluency, cells were concentrated to obtain OD_600_ values of 20, 15, 10, 5, and 1. To assess the relationship between OD_600_ and cell concentration, cultures at each OD_600_ value were serially diluted, and 20 µL of cells from the 10^− 4^, 10^− 5^, and 10^− 6^ dilutions were plated onto YPD agar plates in triplicate. Colony-forming units per milliliter (CFU/mL) were determined and plotted against the OD_600_. A second-order polynomial (quadratic) model was applied to fit the data, yielding R² values of 0.98 and 0.99 for WT and LIP02, respectively. These equations were used to interpolate cell concentrations from OD_600_ values in the cellobiose degradation assay. To evaluate the relationship between OD_600_ and dry cell weight (DCW), overnight cultures of *Sb* MYA 797 and LIP02 were concentrated to OD_600_ values of 20, 15, 10, and 5. One-milliliter aliquots from each OD_600_ condition were collected in pre-weighed tubes, and the supernatant was removed. Cell pellets were dried at 70 °C for 24 h, and dry cell mass was determined by subtracting the pre-weighed tube mass from the total post-drying weight. DCW values were plotted against OD_600_, and the data were fitted using simple linear regression (R^2^ > 0.99). The resulting equations were applied to interpolate DCW from OD_600_ values obtained in the cellobiose consumption assay. The specific cellobiose consumption rate at 24 h was calculated by dividing the cellobiose consumption rate (g/L⋅h) by the corresponding DCW, as determined from OD_600_ measurements at 24 h.

### Growth curve assessment

As illustrated in Fig. S5a, the WT and LIP02 strains were streaked on YPD agar plates. Single colonies were inoculated into YPD medium and incubated overnight at 30 °C. Subsequently, 1 µL of the overnight culture was inoculated into 200 µL of YPD medium in a 96-well plate and incubated at 30 °C with gentle shaking for 48 h using a microplate reader. OD_600_ values were recorded every 15 min, and growth curves were fitted to a Gompertz model using Prism software. Due to suboptimal growth conditions (e.g., small volume and insufficient aeration) in the microplate reader, an alternative method was used to generate the growth curves for WT *int#11::aaBGL* and LIP02 *int#11::aaBGL*. Single colonies were inoculated into YPC medium and incubated overnight at 30 °C. The overnight cultures were reinoculated into fresh YPC medium with an initial OD_600_ of 1. OD_600_ measurements were taken at 0, 12, 24, 36, and 48 h. Growth curves were again fitted to a Gompertz model using Prism.

### Statistical analysis

All statistical analyses were performed using GraphPad Prism (v10.2.2) software. One-way ANOVA was used for multiple-group comparisons, followed by Tukey’s post hoc test to account for multiple comparisons and reduce the risk of Type I error (false positives). A two-tailed t-test was applied for comparisons between two groups. A P value < 0.05 was considered statistically significant.

## Results

### The *Sc* display system is transferable to *Sb*

The Aga2p (NM_001180897.3) and Sed1p (AF510224.1) anchors, widely used in *S. cerevisiae* (*Sc*) display system, were tested in *S. boulardii* (*Sb*) due to their genomic similarity. Co-expression of Aga1p and Aga2p on a plasmid has been reported for *Sb* display [21]; however, such large plasmids may reduce transformation efficiency [38] and limit heterologous protein expression [39]. To address this, we implemented an Aga2-based system where plasmid-expressed Aga2 bound to chromosomally encoded Aga1 via disulfide bonds. For the Sed1-based system, the Sed1 anchor was amplified from *Sb* MYA 797 and integrated into a display plasmid (Fig. S1a).

*Sb* display capability was evaluated using two parameters: expression level and display level. The intensity of yeGFP was used to assess cassette expression, while Alexa Fluor^®^ fluorescence (via an anti-myc antibody, Invitrogen MA1-980, or an anti-HA antibody, Thermo Fisher #26183-A555) was detected by flow cytometry to measure display levels (Fig. S1a). Flow cytometry results indicated that *Sc* outperformed *Sb* in display efficiency for both Aga2- and Sed1-based systems. *Sc* exhibited higher plasmid expression, as shown by stronger mean fluorescence intensity (MFI) of yeGFP in the Aga2 system (Fig. S1b and c). For the Sed1 system, *Sc* and *Sb* had similar expression levels, but overall MFI was lower compared to the Aga2 system, indicating reduced expression and display efficiency. These results validated the design of the display cassette and demonstrated that both Aga2- and Sed1-based display systems were compatible with *Sb*.

### Optimization of the *Sb* display cassette

Previous studies have explored strategies to enhance display efficiency by optimizing cassette components, including anchor proteins [22], anchor length [27], secretion peptides [31, 32], and promoters [25]. Building on these findings, we optimized the display cassette to improve *Sb* display efficiency.

A panel of plasmids carrying a *URA3* auxotrophic marker and various display cassettes (Fig. 1a) was constructed and transformed into *Sb* MYA 797 *ura3Δ*. For the Aga2-Aga1 system, two plasmids were designed: A1 and A2, differing only by the presence of a G4S linker between Aga2 and yeGFP. Based on flow cytometry analysis, A2, which included the linker, exhibited significantly higher MFI than A1, indicating that the linker enhanced expression and display levels (Fig. 1b).

For the Sed1-based display system, we evaluated the impact of anchor length. While previous studies in *S. cerevisiae* (*Sc*) identified Flo428p, the 428-amino-acid C-terminal region of Flo1p, as an optimal anchor [27], the low sequence similarity (< 10% at the protein level) between *Sc*Flo1p and *S. boulardii* (*Sb*) Flo1p suggests this optimization may not be applicable to *Sb*. Given the high similarity between *Sc*Sed1p and *Sb*Sed1p, we modified the length of *Sb*Sed1p, a glycosylphosphatidylinositol (GPI)-dependent anchor, to assess its impact on display efficiency. Sed1p is a widely used anchor for *Sc* and *Pichia pastoris* (*Pp*) display systems, and studies have utilized different Sed1p lengths, including full-length Sed1p in *Sc* [40] and *Pp* [26], Sed1p without its native signal peptide in *Pp* [41], and truncated (227 amino acids) Sed1p containing only the C-terminal anchoring region in *Sc* [22, 42]. Since no direct comparisons between these variations exist, we tested different lengths of *Sb*Sed1p. To avoid potential secretion peptide cleavage causing POI detachment [43], we tested *Sb*Sed1p without its native signal peptide (Sed1 anchor) and a shorter version containing only the C-terminal anchor region (Sed1 anchor (p)), represented as S1–S4 in Fig. 1a. The results indicated that full-length *Sb*Sed1p provided significantly better display efficiency compared to the truncated anchor (Fig. 1b).

We also examined two secretion peptides: the native signal peptide of *Sb*Sed1p (Sed1sp) and the mating factor α secretion peptide (MFαsp). While no significant differences were observed between the two when using the truncated *Sb*Sed1p, MFαsp conferred significantly higher display efficiency than Sed1sp when paired with full-length *Sb*Sed1p (Fig. 1b). Based on these results, A2 and S1 were selected as the final display cassettes for the Aga2- and Sed1-based display systems, respectively, for subsequent experiments due to their superior expression and display levels.

### Host modification to enhance display capability

Host modifications have been demonstrated to augment display efficiency by expanding cell wall capacity to accommodate more POIs [26, 44] or by improving protein secretion to facilitate the availability of POIs for display [42, 45]. To address the first rationale, chromosomal *AGA1* (KF369586) was overexpressed by replacing the native promoter with the strong constitutive *GPD* (*TDH3*) promoter, which has been validated in *Sb* for robust expression [15, 36]. This strategy was inspired by *Sc* EBY100 strain, where chromosomal *AGA1* was overexpressed under a galactose-inducible promoter instead of the native promoter [46]. Overexpressing *AGA1* can provide more docking sites for Aga2p to enhance POI display.

**Fig. 2:**
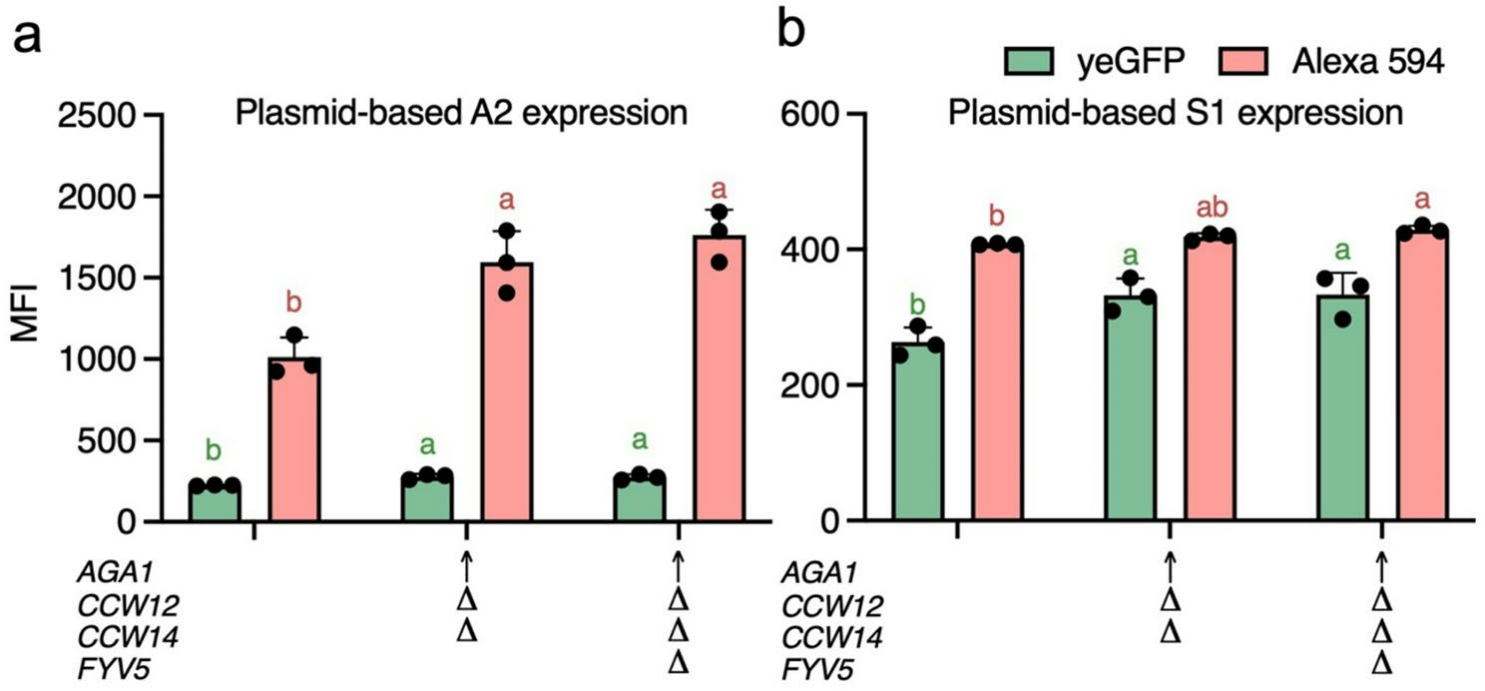
Evaluation of host strain modifications for protein display efficiency. (**a**) Flow cytometry analysis of *Saccharomyces boulardii* (*Sb*) transformed with a plasmid expressing A2. (**b**) Flow cytometry analysis of *Sb* transformed with a plasmid expressing S1. Mean fluorescence intensity (MFI) values were measured for yeast-enhanced GFP (yeGFP) and Alexa 594 signals to assess protein display efficiency. Error bars indicate standard deviation (*n* = 3). Statistical significance was evaluated using one-way ANOVA (*p* < 0.05), with different letters denoting significant differences between groups. Host strain modifications are labeled below the x-axis as follows: ↑ indicates overexpression, and Δ indicates deletion of the targeted gene

Additionally, *CCW12* (NM_001181997.1) and *CCW14* (NM_001184303.1) were targeted to improve display efficiency, as their co-deletion in *Sc* has been shown to increase cell wall thickness [44]. The strain *Sb* MYA 797 *ura3Δ TDH3*_*p*_*-AGA1 ccw12Δ ccw14Δ* (LIP01) was constructed and transformed with the plasmid expressing A2 (Fig. 1a). Compared to the parental strain (*Sb* MYA 797 *ura3*Δ expressing A2), LIP01 exhibited significantly higher display levels as indicated by increased MFI (Fig. 2a).

To further enhance display efficiency, *FYV5* (NM_001178702.1) was selected as an additional target. In *Sc*, co-deletion of *CCW12* and *FYV5* upregulated genes involved in protein processing in the endoplasmic reticulum (ER) and protein translation [42]. Furthermore, improved protein transportation led to better display efficiency in *Sc* [45]. The strain MYA 797 *ura3Δ TDH3*_*p*_*-AGA1 ccw12Δ ccw14Δ fyv5Δ* (LIP02) was constructed and transformed with the plasmid expressing A2 (Fig. 1a and Fig. S2b). PCR confirmation of the genotype is shown in Fig. S2a. LIP02 also exhibited significantly higher display levels compared to the parental strain (Fig. 2a). However, no significant difference was observed between LIP01 and LIP02 in the context of the Aga2*-*based system.

To evaluate the compatibility of these host modifications with the Sed1-based display system, a plasmid expressing S1 was transformed into the control strain (*Sb* MYA 797 *ura3Δ*), LIP01, and LIP02 (Fig. 1a and Fig. S2b). LIP02 demonstrated significantly higher display levels compared to the control strain (Fig. 2b). However, as observed previously (Fig. S1c), the Sed1-based system showed lower expression and display levels compared to the Aga2-based system (Fig. 2a and b), differing from findings in *Sc* [22]. Given its enhanced performance with both Aga2- and Sed1-based display systems, LIP02 was selected as the host strain for subsequent experiments. Additionally, the Aga2-based configuration was chosen for demonstrating *Sb* display due to its superior display levels.

Beyond enhancing display efficiency, host modifications in LIP02 also led to physiological changes. Forward scatter (FSC) analysis in flow cytometry indicated that LIP02 cells were larger than the parental strain (Fig. S2c). To further investigate this, we examined the relationship between OD_600_ and both dry cell weight (DCW) and colony-forming unit (CFU) counts. At the same value of OD_600_, LIP02 exhibited a lower DCW (Fig. S3a) and a reduced concentration (CFU/mL) compared to the parental strain (Fig. S3b), suggesting altered cell density. We accounted for these physiological differences in subsequent experiments to ensure fair comparisons of display efficiency and enzymatic activity.

### Genome integration of the display cassette into LIP02

**Fig. 3:**
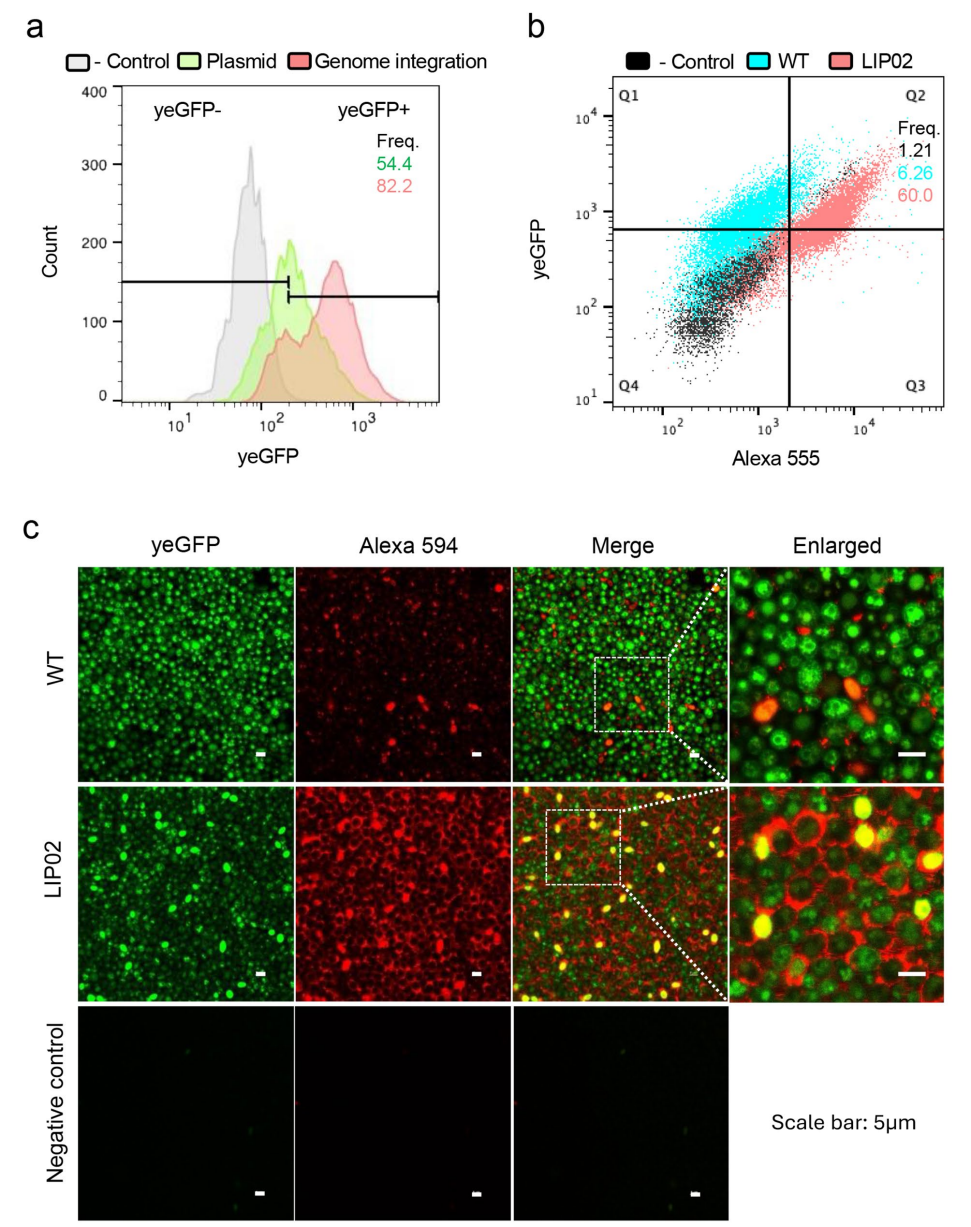
Comparative analysis of plasmid-based and genome-integrated display systems. (**a**) Flow cytometry comparison of *Saccharomyces boulardii* (*Sb*) strains expressing A2 through plasmid-based (green) or genome-integrated (red) systems. Frequency (Freq.) represents the percentage of yeGFP-positive (yeGFP+) events gated using a negative control (parental *Sb* strain labeled with antibodies). (**b**) Flow cytometry comparison of wild-type (WT) *Sb* and LIP02 strains containing the genome-integrated A2 cassette. Events in quadrant Q2 (Alexa 555-positive, yeGFP-positive) are color-coded to emphasize differences in display efficiency. Frequencies were calculated based on 10,000 cells analyzed per sample. (**c**) Confocal microscopy images of WT and LIP02 strains containing genome-integrated A2 and labeled with Alexa 594 antibody. The yeGFP signal (green) and Alexa 594 signal (red) indicate successful expression and display of the A2 cassette. Negative controls include the parental strain (WT *Sb*) subjected to identical labeling and imaging conditions. White boxes highlight regions of interest, with zoomed-in views shown on the right

Plasmid-based expression systems require selective media for cultivation, which may lead to population heterogeneity due to cross-feeding of metabolites, as demonstrated in a recent study [47]. To evaluate plasmid stability, LIP02 harboring the plasmid expressing A2 was passaged five times in YPD medium without selective pressure. Randomly selected single colonies were then streaked onto SC-ura agar, where most failed to grow (Fig. S4a). These results indicate that the plasmid expression system is unstable. To develop a more stable yeast population capable of growing in non-selective media, the display cassette A2 (Fig. 1a) was integrated into the intergenic region of the WT strain (*Sb* MYA 79*7 ura3Δ*) and LIP02. Successful integration was confirmed via PCR (Fig. S4b), and the stability of the genome-integrated A2 cassette was verified after five serial transfers in YPD (Fig. S4a). Flow cytometry analysis revealed that genome-integrated LIP02 exhibited a larger population of yeGFP positive cells compared to plasmid-carrying LIP02 (Fig. 3a). This may suggest plasmid loss in part of the population, potentially due to cross-feeding during the 48-hour cultivation in SC-ura medium prior to analysis. Genome-integrated LIP02 also displayed significantly higher display levels compared to genome-integrated WT (Fig. 3b), underscoring the enhanced display capacity of LIP02 when the display cassette was chromosomally integrated.

To visualize the improved display ability of LIP02, genome-integrated WT and LIP02 strains were examined using confocal microscopy. Antibody labeling highlighted the yeast cell surface, showing well-defined cell morphology in LIP02 compared to the less distinct outlines of WT (Fig. 3c). This observation further supports the enhanced display capability of LIP02. Interestingly, a reduced yeGFP signal was observed in LIP02 compared to WT, where the majority of the yeGFP fluorescence originated intracellularly. This difference may be attributed to the spatial distribution of yeGFP. Exposure of GFP to the extracellular environment likely reduces its fluorescence intensity due to environmental factors such as pH [48]. Despite this, confocal microscopy and flow cytometry results collectively confirmed the enhanced display efficiency of LIP02 following genome integration of the display cassette.

### Engineered *Sb* strain maintains enzyme functionality

**Fig. 4:**
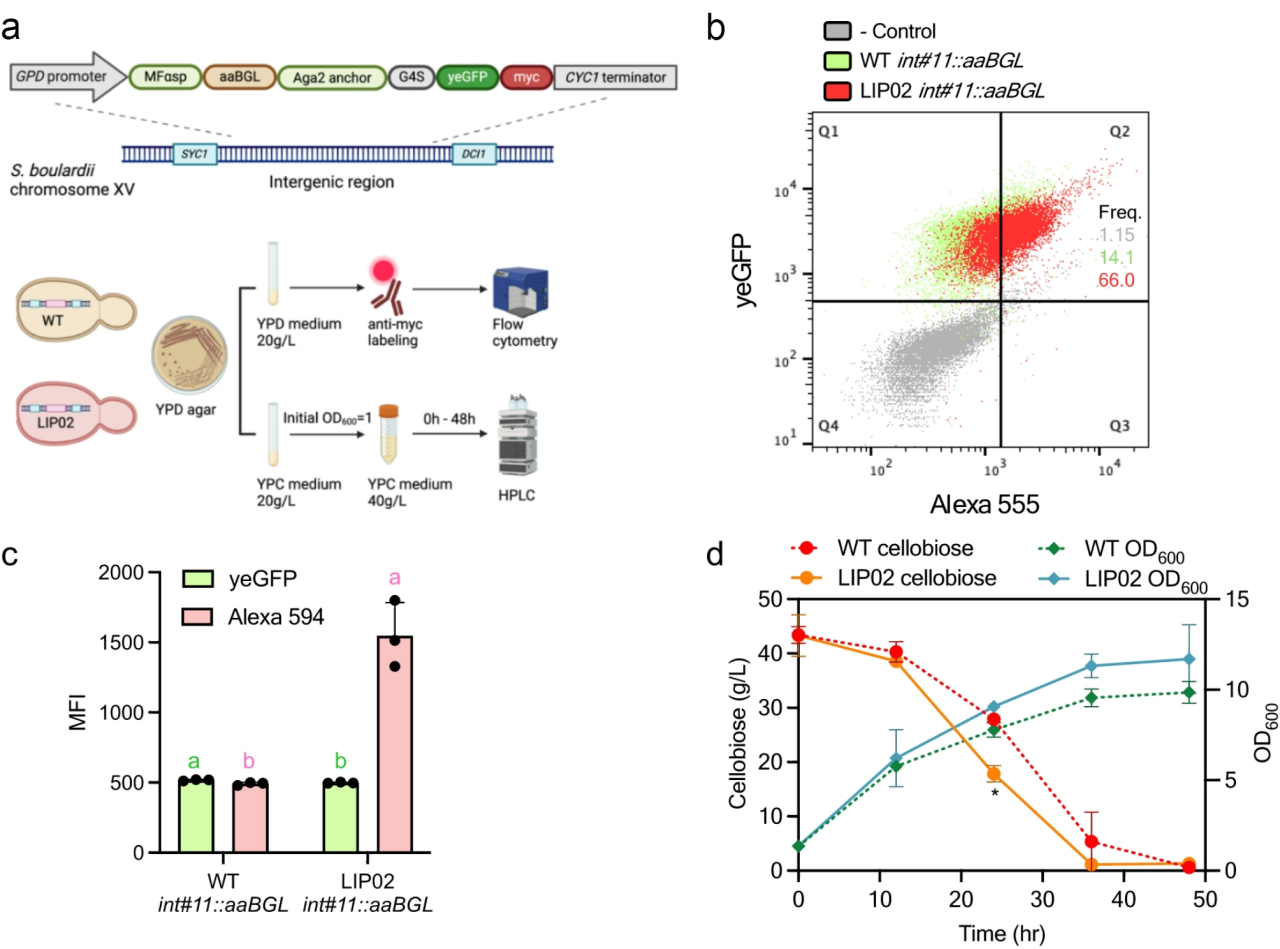
Functional assessment of β-glucosidase (*aaBGL*) display in *Saccharomyces boulardii*. (**a**) Schematic representation of the experimental workflow for evaluating wild-type (WT) and engineered (LIP02) *S. boulardii* strains with a genome-integrated *aaBGL* display cassette at *int#11*. The workflow includes labeling with Alexa Fluor^®^ antibody for flow cytometry analysis and cellobiose degradation assays measured by HPLC. (**b**) Flow cytometry analysis showing the distribution of Alexa 555 (anti-HA labeling) and yeGFP signals. Quadrant Q2 (top-right) represents cells displaying both signals. Frequencies (Freq.) denote the percentage of events in Q2 out of 10,000 cells: LIP02 (66.0%) and WT (14.1%). (**c**) Quantitative representation of mean fluorescence intensity (MFI) from flow cytometry analysis. Bars indicate MFI of Alexa 594 in WT and LIP02 strains. Different letters indicate statistically significant differences (*p* < 0.05). (**d**) Cellobiose degradation and growth analysis. The left y-axis shows cellobiose consumption (measured by HPLC), and the right y-axis represents cell growth (OD_600_) over 48 h. Data highlight enhanced cellobiose utilization and growth in the LIP02 strain compared to WT

To assess whether host modifications affected the functionality of the displayed POI, the activity of β-glucosidase was evaluated when displayed on the surface of LIP02. Native *Sb* cannot ferment or utilize cellobiose as its sole carbon source [49, 50]. Cell growth is only enabled by the hydrolysis of cellobiose into glucose, facilitated by heterologous β-glucosidase, allowing the cells to consume glucose. Since genome integration provided strain stability without compromising display capability, an Aga2-based display cassette containing β-glucosidase (841 amino acids without the native signal peptide) from *A. aculeatus* (*aaBGL*) was integrated into the intergenic region *int#11* of both WT and LIP02 (Fig. 4a). Previous studies have demonstrated that fusing the C-terminal of β-glucosidase to the N-terminal of an anchor protein preserved enzymatic activity [25, 51]. To facilitate proper enzymatic function, *aaBGL* was fused to the N-terminal of Aga2, while yeGFP was placed at the C-terminal (Fig. 4a). This configuration, also tested in recent work, allows the POI’s N-terminal to interact with ligands [32]. The integrated strains were analyzed using flow cytometry to measure display levels and a cellobiose degradation assay to evaluate β-glucosidase activity.

LIP02 with genome-integrated *aaBGL* showed a higher percentage of fluorescence-positive cells compared to WT (Fig. 4b). The higher MFI of Alexa 555, but not yeGFP, in LIP02 indicated that more *aaBGL* was displayed without an increase in display cassette expression (Fig. 4c). This correlated with faster cellobiose degradation by *aaBGL* in LIP02 from 12 to 36 h compared to WT (Fig. 4d). Based on the relationship between OD_600_ and dry cell weight (DCW) (Fig. S3a) and between OD_600_ and CFU concentration (Fig. S3b), LIP02 exhibited lower DCW and CFU at the same OD_600_ compared to WT, indicating changes in cellular morphology. Despite these physiological differences, LIP02 showed a twofold increase in specific cellobiose degradation rate at 24 h, based on DCW (Table S4). The growth curve of each strain, assessed by OD_600_, was also analyzed. Growth curves of WT and LIP02 without *aaBGL* integration under glucose conditions, fitted to a Gompertz model, showed similar fitness in YPD medium (Fig. S5). However, under cellobiose conditions, WT and LIP02 with *aaBGL* integration demonstrated the effect of display capability on specific cellobiose consumption rates (Fig. 2d). Given the lower cell concentration of LIP02 at the same OD_600_ as WT, the data suggest that the faster degradation of cellobiose is due to an increased display of *aaBGL* rather than a higher cell count. These findings further confirm LIP02’s superior display capability.

## Discussion

To create a probiotic yeast strain with enhanced display capabilities, this study employed two strategies: optimizing the display cassette and modifying the host strain. Key factors influencing display efficiency in the display cassette include the choice of anchor type [22], anchor length [27], signal peptide [31–33], and linker design [22, 32]. In this study, the Aga2 anchor demonstrated superior expression and display levels compared to the Sed1 anchor (Fig. 1b and Fig. S1c), contrary to observations in *Sc* [22]. The observed differences between Aga2-based and Sed1-based display systems, as well as variations between *Sc* and *Sb*, may arise from differences in anchor protein interactions with the cell wall. Genomic variations between *Sc* and *Sb* include differences in transposable elements, sugar transporters, and flocculin gene copy number [52], as well as differential gene expression due to promoter variability [19]. Minor genomic changes have been shown to alter protein secretion, as reported in *Komagataella phaffii* (formerly *Pichia pastoris*) [53] and *Sc* [54]. Additionally, the cell wall composition of *Sb* differs from that of *Sc*, with *Sb* exhibiting a higher mannoprotein content and lower glucan levels [55, 56]. Since *SED1* encodes a glycosylphosphatidylinositol (GPI)-anchored protein covalently linked to β-1,3-glucan in the inner cell wall layer, and Aga2 binds via disulfide interactions with Aga1, differences in localization may influence the accessibility of displayed proteins in *Sb*. Consequently, display efficiency depends on both the choice of anchor protein and the host strain. Furthermore, the impact of the signal peptide on the Sed1-based configuration in *Sb* was found to be minimal. Previous findings in *Sc* reported that the *Sc*Sed1 signal peptide enhanced the display and secretion of cellulolytic enzymes compared to the *Rhizopus oryzae* glucoamylase signal peptide (GLUASP) and the *Sc* α-mating pheromone signal peptide (MFα1SP) [31]. However, in the *Sb*Sed1-based system, Sed1SP performed similarly or was occasionally inferior to MFα1SP (Fig. 1b). Two lengths of Sed1p were evaluated as both have been utilized for surface display in *Sc* and *Pp* [22, 26, 40–42, 57], but no comparative analysis has been previously conducted. In *Sb*, the original length of Sed1p (excluding its native signal peptide) conferred higher display levels (Fig. 1b). The full-length anchor may provide greater flexibility, improving accessibility to the displayed protein and enhancing its presentation. This flexibility, essential for optimizing protein exposure, is further influenced by linker sequences. Linkers enable independent movement of protein domains, reducing steric hindrance and promoting proper folding. Chen et al. reported that flexible linkers such as G4S enhance the biological activity and stability of fusion proteins by minimizing unfavorable domain interactions. This flexibility facilitates proper folding and increases the surface exposure of functional sites, which is crucial for optimal fusion protein performance [58]. In this study, display cassettes containing a Gly and Ser repeating linker (G4S) exhibited enhanced display efficiency in the WT *Sb* strain compared to cassettes lacking linkers (Fig. 1b), consistent with findings in *Sc* [22]. This enhancement may be attributed to the G4S linker’s ability to reduce steric interference and promote proper protein folding and surface exposure, as reported in previous studies [58–60].

Host strain modification was pursued to further enhance display capabilities, leading to the development of the LIP02 strain. These modifications were designed with specific mechanistic advantages to improve protein display. A key strategy involved the overexpression of *AGA1* in the Aga2p-Aga1p display system. The native promoter was replaced with the strong, constitutive *GPD* (*TDH3*) promoter, which has been shown to drive robust expression in *Sb* [15]. The overexpression of *AGA1* increased the number of docking sites. This directly contributed to higher levels of POI presentation on the cell surface. In addition, the deletion of *CCW12* and *CCW14*, which encode GPI-anchored cell wall proteins, aimed to improve the cell wall accessibility. These genes are involved in cell wall organization, and their deletion increases cell size (Fig. S2c), providing more space for protein anchoring. By knocking out these genes, we anticipated enhanced display efficiency due to the increased surface area available for protein attachment. A similar strategy was employed in a previous study. The disruption of *SED1* in *Sc* resulted in increased surface display by freeing up cell wall space for enzyme display [26]. Future study could explore this approach to further enhance *Sb* display. Furthermore, the deletion of *FYV5* was included due to its role in upregulating protein processing in the ER and enhancing protein transport from the ER to the Golgi. These steps are essential for efficient secretion and surface display [43]. Studies have demonstrated that upregulation of protein translation and folding pathways, as well as improving protein trafficking, significantly boosts secretion and surface-display capabilities [42]. Taken together, these modifications were expected not only to increase the available docking sites for POIs but also to optimize the overall protein secretion and display process. The improved performance of LIP02 in our study provided strong evidence supporting this optimization.

LIP02 demonstrated improved display efficiency in both Aga2- and Sed1-based systems (Fig. 2), as well as plasmid-based and genome-integrated expression settings (Fig. 3). Recent studies have highlighted cross-feeding within yeast populations, which generates metabolic heterogeneity [47] and metabolites exchange, which fosters obligatory syntrophic relationships [61] in selective media. In this study, plasmid-carrying strains depended on the *URA3* selective marker and SC-ura medium for plasmid maintenance. The interoperation of fluorescent-positive populations and MFI was based on the assumption that all strains can maintain the plasmid equally. However, a lower fluorescent-positive population or reduced MFI could indicate decreased plasmid retention (Fig. S4a), possibly due to metabolite exchanges or cross-feeding within the population [47]. Genome integration of the display cassette circumvents this issue, yielding a more stable strain capable of growing in complex medium (Fig. S4a). As shown in Figs. 3 and 4, genome-integrated LIP02 exhibited significantly enhanced display efficiency relative to WT strains, without compromising protein functionality. These results underscore the success of host strain modification in achieving robust display capabilities.

A major challenge in using genetically modified probiotic strains lies in the regulatory constraints, as stringent guidelines govern their applications, particularly in therapeutics [62]. Integrating stable genome modifications in *Sb* may aid regulatory approval by minimizing plasmid-related concerns such as horizontal gene transfer of antibiotic markers. To promote real-world applicability, future research should prioritize evaluating the in vivo stability of this strain. Preliminary studies have assessed *Sb* in this context. Heavey et al. demonstrated the stability of Aga2-based display in simulated intestinal fluid and in vivo [63], while Sands et al. characterized promoter strength across diverse environments, including varying oxygen levels, carbon sources, and in vivo conditions [64]. Collectively, these studies, along with the present work, provide a foundation for the development of robust therapeutic strains.

The impact of host modifications on cell physiology was also assessed. Consistent with previous studies in *Sc*, manipulation of cell wall proteins increased cell size, providing more docking space for the displayed protein. Due to this size change, LIP02 exhibited lower DCW and cell concentration at the same OD_600_ value compared to the WT. However, despite the reduced cell count, LIP02 displaying *aaBGL* consumed cellobiose more rapidly (Table S4), confirming that its enhanced display capacity resulted in a higher amount of displayed enzyme.

## Conclusion

Yeast display systems exhibit remarkable versatility and have gained prominence across various industries. While *Sc* and *Pp* are commonly utilized in biorefinery applications with well-optimized systems, *Sb*, a superior host for biomedical and clinical applications due to its probiotic and prebiotic properties, lacks a well-characterized display platform. Despite numerous studies on the development and characterization of bioengineered *Sb* strains, efforts to optimize its display system have been limited. In this study, a CRISPR-Cas9-based strategy was employed to construct an *Sb* strain with enhanced display capabilities. This strain was evaluated using multiple parameters, including protein expression levels, display efficiency, functionality of the displayed protein, and host fitness. The results demonstrated that the newly engineered *Sb* strain is highly suitable for the display of large heterologous proteins, maintaining protein functionality. However, we observed physiological changes in LIP02, including increased cell size and altered biomass-to-OD ratios. While this study did not assess the impact of these modifications under industrial or therapeutic conditions, our findings provide a foundation for future investigations into the strain’s robustness and application potential in diverse environments. Future research should evaluate the strain’s stability under various fermentation conditions, including pH fluctuations, oxygen levels, and nutrient availability, to mimic the stress LIP02 encounters during passage through the digestive tract. Since the host modification might impact the cell wall structure, in vivo studies are needed to examine the impact on immunogenicity and therapeutic efficacy. Such studies will provide a comprehensive understanding of the strain’s potential benefits and limitations, guiding future translational efforts toward clinical applications.

## Electronic supplementary material

Below is the link to the electronic supplementary material.

Supplementary Material 1

## Data Availability

No datasets were generated or analysed during the current study.

## Abbreviations

*Sb*: *Saccharomyces boulardii*
*Sc*: *Saccharomyces cerevisiae*
*Pp*: *Pichia pastoris*
SP: Signal peptide
WT: Wild-type
POI: Protein of interest
ORF: Open reading frame
dsDNA: Double strand DNA
PCR: Polymerase chain reaction
CRISPR: Clustered regularly interspaced short palindromic repeats
YPD: Yeast extract peptone dextrose
YPC: Yeast extract peptone cellobiose
LB: Luria-Bertani
aaBGL: β-glucosidase from *Aspergillus aculeatus*
