# Supplementary material for: Engineering Saccharomyces boulardii for enhanced surface display capacity
Source: Microb Cell Fact. 2025 Apr 1;24:76. doi: 10.1186/s12934-025-02702-3 (PMC11959792; doi:10.1186/s12934-025-02702-3)
Supplement: Supplementary file 1 — Supplementary Material 1 [file 12934_2025_2702_MOESM1_ESM.docx]

**Supplementary information**

**Engineering *Saccharomyces boulardii* for Enhanced Surface Display Capacity**

Luping Xu^1,2^, Xingjian Bai^3^, Deokyeol Jeong^1,2^, Dahye Lee^1,2^, Fransheska Semidey^1,2^, Chenhai Li^1,2^, Eun Joong Oh^1,2^

^1^Department of Food Science, Purdue University, West Lafayette, IN 47907, USA

^2^Whistler Center for Carbohydrate Research, Purdue University, West Lafayette, IN 47907, USA

^3^Vectorbuilder Inc., Chicago, IL 60609, USA

Correspondence should be addressed to Eun Joong Oh (ejoh@purdue.edu).

a


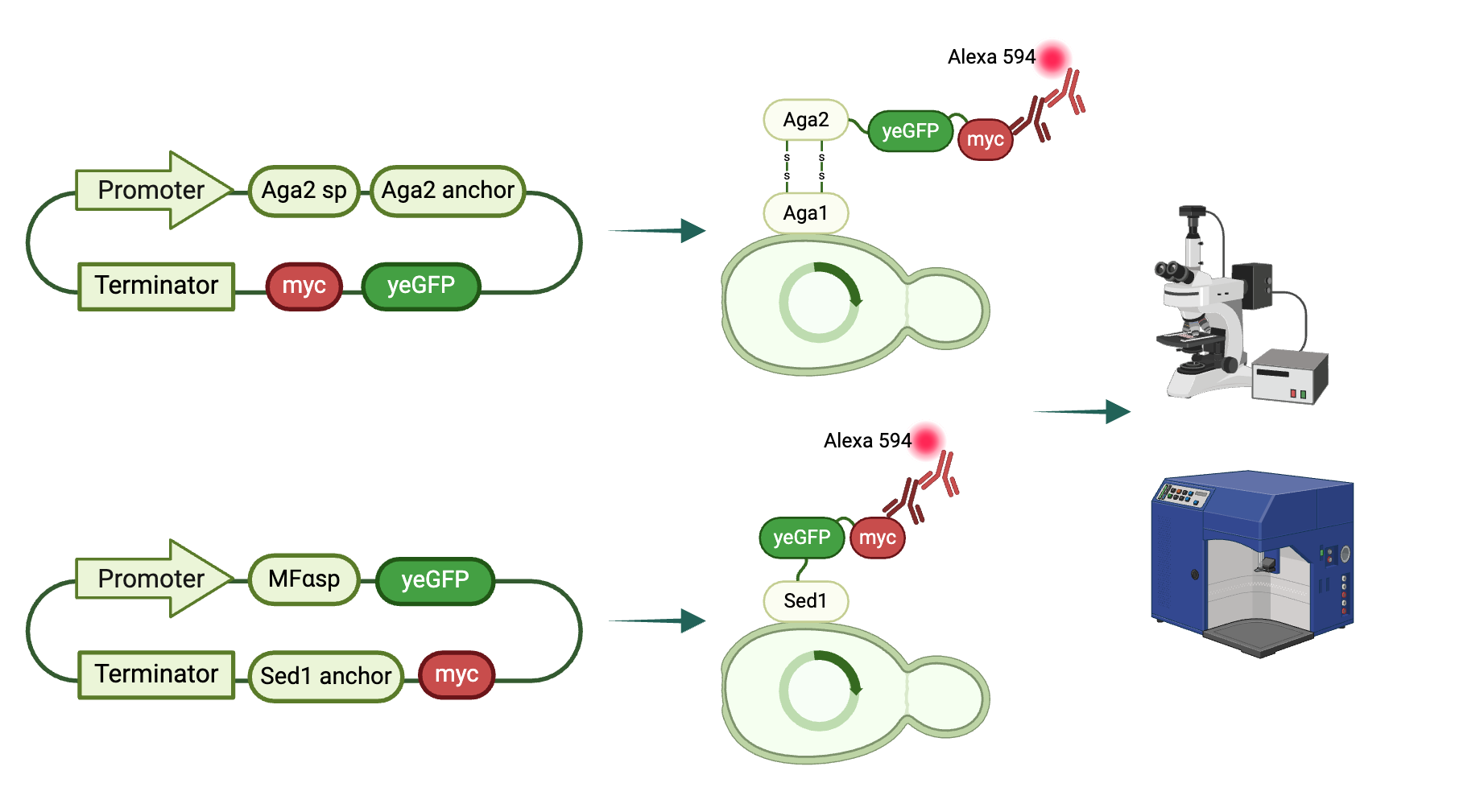


b

c

Figure S1. Plasmid-based surface display systems. (a) Yeast display characterization procedures. (b) Fluorescent microscopic images of *Sc* and *Sb* carrying different display plasmids. (c) Mean fluorescence intensity (MFI) of Alexa-594 in *Sc* and *Sb* analyzed using flow cytometry.


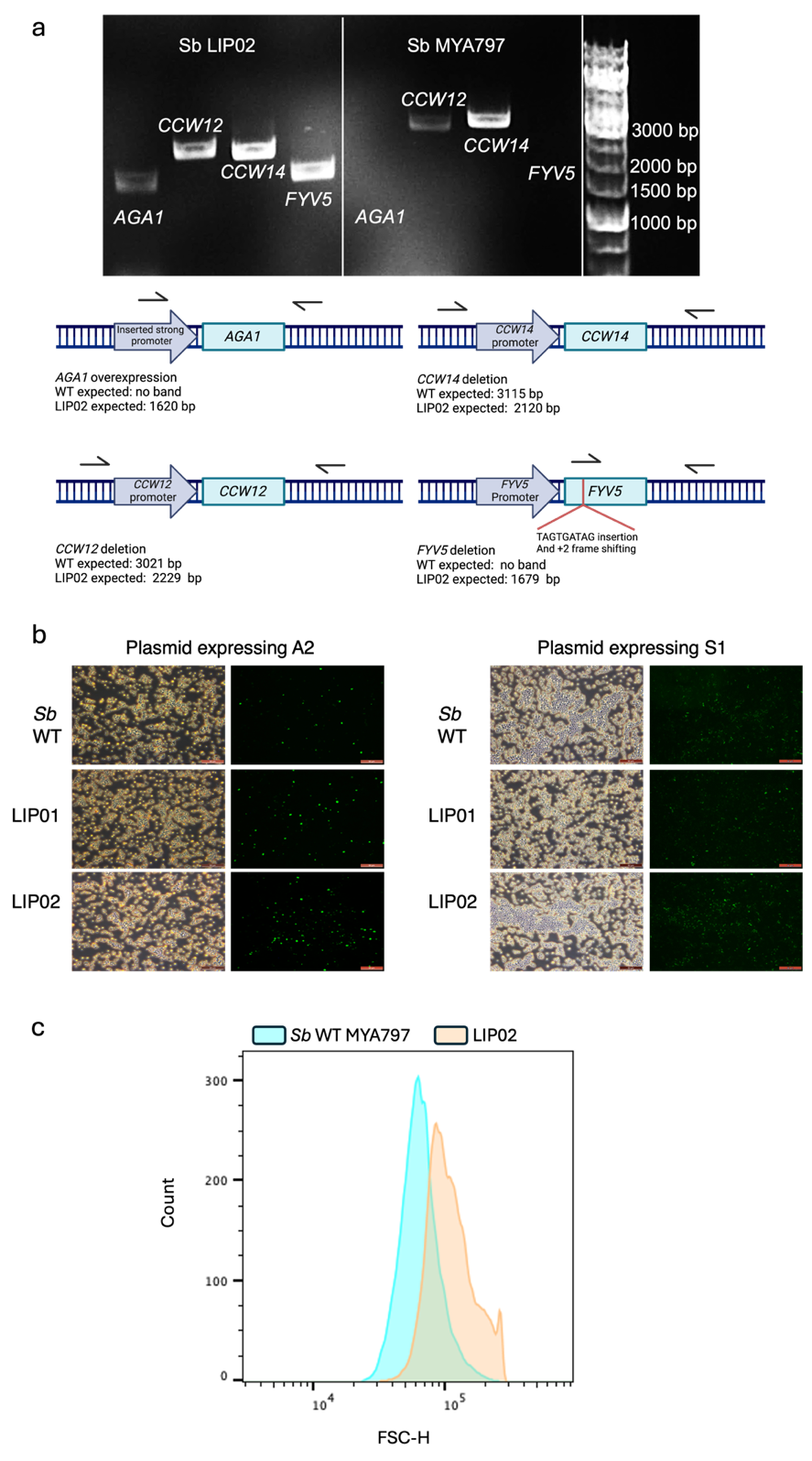


Figure S2. Strain construction and confirmation. (a) PCR confirmation of the LIP02 genotype. Primer binding locations are shown in the bottom panel. (b) Fluorescent microscopic images of *Sb* transformed with plasmid expressing A2 and S1. (c) Forward scatter (FSC) analysis of wild-type (WT) and LIP02 strains to assess cell size distribution. WT (blue) and LIP02 (orange) are shown, with a total of 10,000 events recorded for each sample.

Figure S3. Analysis of the relationship between optical density (OD) and biomass/cell concentration. (a) Correlation between OD and dry cell weight (DCW, g/L). Linear regression equations and R² values indicate a strong relationship, enabling the estimation of cell biomass based on OD measurements. (b) Relationship between OD and cell concentration (CFU/mL), illustrating how OD correlates with cell density. Data were fitted using a second-order polynomial (quadratic) model. WT: *Saccharomyces boulardii* MYA-797 wild-type; LIP02: strain overexpressing *AGA1* and deleting cell wall-related genes (*CCW12, CCW14,* and *FYV5*).

Figure S4. Plasmid carrying strain and genome integrated strain characterization. (a) Left panel: plasmid stability assay after five serial subcultures in YPD medium. Top plate: YPD agar, demonstrating overall cell growth. Bottom plate: SC-ura agar, selecting for plasmid retention. Right panel: colony PCR analysis of the genome-integrated expression cassette in LIP02 subcultures 1 and 5 after five serial passages in YPD medium. (b) Genome integration of the display cassette. PCR confirmation of the display cassette A2 integrated into the genome of LIP02 and WT strains. The right panel shows LIP02 without genome integration as a negative control. The bottom panel illustrates the genome insertion site and primer binding locations.

a


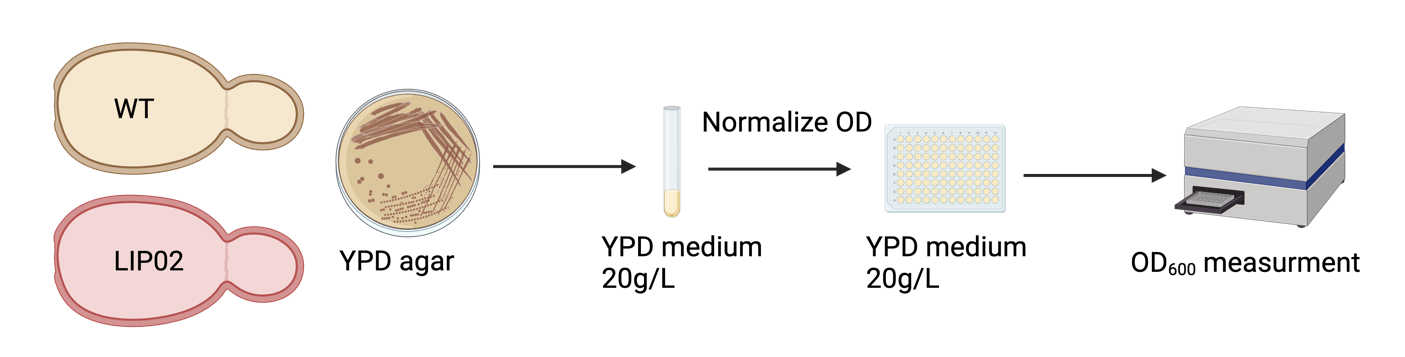


b

Figure S5. Growth curve assessment. (a) Schematic illustration of the OD_600_ measurement for LIP02 and WT lacking the *aaBGL* display cassette. (b) Growth curves were fitted to a Gompertz model using Prism software.

Table S1. Plasmid open reading frames (ORFs) and backbone sequences

| Display cassette | ORF sequence |
| --- | --- |
| A1 | ATGCAGTTACTTCGCTGTTTTTCAATATTTTCTGTTATTGCTTCAGTTTTAGCACAGGAACTGACAACTATATGCGAGCAAATCCCCTCACCAACTTTAGAATCGACGCCGTACTCTTTGTCAACGACTACTATTTTGGCCAACGGGAAGGCAATGCAAGGAGTTTTTGAATATTACAAATCAGTAACGTTTGTCAGTAATTGCGGTTCTCACCCCTCAACAACTAGCAAAGGCAGCCCCATAAACACACAGTATGTTTTTAAGGACAATAGCTCGACGATGTCTAAAGGTGAAGAATTATTCACTGGTGTTGTCCCAATTTTGGTTGAATTAGATGGTGATGTTAATGGTCACAAATTTTCTGTCTCCGGTGAAGGTGAAGGTGATGCTACTTACGGTAAATTGACCTTAAAATTTATTTGTACTACTGGTAAATTGCCAGTTCCATGGCCAACCTTAGTCACTACTTTCGGTTATGGTGTTCAATGTTTTGCTAGATACCCAGATCATATGAAACAACATGACTTTTTCAAGTCTGCCATGCCAGAAGGTTATGTTCAAGAAAGAACTATTTTTTTCAAAGATGACGGTAACTACAAGACCAGAGCTGAAGTCAAGTTTGAAGGTGATACCTTAGTTAATAGAATCGAATTAAAAGGTATTGATTTTAAAGAAGATGGTAACATTTTAGGTCACAAATTGGAATACAACTATAACTCTCACAATGTTTACATCATGGCTGACAAACAAAAGAATGGTATCAAAGTTAACTTCAAAATTAGACACAACATTGAAGATGGTTCTGTTCAATTAGCTGACCATTATCAACAAAATACTCCAATTGGTGATGGTCCAGTCTTGTTACCAGACAACCATTACTTATCCACTCAATCTGCCTTATCCAAAGATCCAAACGAAAAGAGAGACCACATGGTCTTGTTAGAATTTGTTACTGCTGCTGGTATTACCCATGGTATGGATGAATTGTACAAAGAGCAAAAGCTCATTTCTGAAGAGGACTTGTAA |
| A2 | ATGCAGTTACTTCGCTGTTTTTCAATATTTTCTGTTATTGCTTCAGTTTTAGCACAGGAACTGACAACTATATGCGAGCAAATCCCCTCACCAACTTTAGAATCGACGCCGTACTCTTTGTCAACGACTACTATTTTGGCCAACGGGAAGGCAATGCAAGGAGTTTTTGAATATTACAAATCAGTAACGTTTGTCAGTAATTGCGGTTCTCACCCCTCAACAACTAGCAAAGGCAGCCCCATAAACACACAGTATGTTTTTAAGGACAATAGCTCGACGATTGAAGGTAGATACCCATACGACGTTCCAGACTACGCTCTGCAGGCTAGTGGTGGAGGAGGCTCTGGTGGAGGCGGTAGCGGAGGCGGAGGGTCGTCTAAAGGTGAAGAATTATTCACTGGTGTTGTCCCAATTTTGGTTGAATTAGATGGTGATGTTAATGGTCACAAATTTTCTGTCTCCGGTGAAGGTGAAGGTGATGCTACTTACGGTAAATTGACCTTAAAATTTATTTGTACTACTGGTAAATTGCCAGTTCCATGGCCAACCTTAGTCACTACTTTCGGTTATGGTGTTCAATGTTTTGCTAGATACCCAGATCATATGAAACAACATGACTTTTTCAAGTCTGCCATGCCAGAAGGTTATGTTCAAGAAAGAACTATTTTTTTCAAAGATGACGGTAACTACAAGACCAGAGCTGAAGTCAAGTTTGAAGGTGATACCTTAGTTAATAGAATCGAATTAAAAGGTATTGATTTTAAAGAAGATGGTAACATTTTAGGTCACAAATTGGAATACAACTATAACTCTCACAATGTTTACATCATGGCTGACAAACAAAAGAATGGTATCAAAGTTAACTTCAAAATTAGACACAACATTGAAGATGGTTCTGTTCAATTAGCTGACCATTATCAACAAAATACTCCAATTGGTGATGGTCCAGTCTTGTTACCAGACAACCATTACTTATCCACTCAATCTGCCTTATCCAAAGATCCAAACGAAAAGAGAGACCACATGGTCTTGTTAGAATTTGTTACTGCTGCTGGTATTACCCATGGTATGGATGAATTGTACAAAGAGCAAAAGCTCATTTCTGAAGAGGACTTGTAA |
| S1 | ATGAGATTTCCTTCAATTTTTACTGCAGTTTTATTCGCAGCATCCTCCGCATTAGCTGCTCCAGTCAACACTACAACAGAAGATGAAACGGCACAAATTCCGGCTGAAGCTGTCATCGGTTACTTAGATTTAGAAGGGGATTTCGATGTTGCTGTTTTGCCATTTTCCAACAGCACAAATAACGGGTTATTGTTTATAAATACTACTATTGCCAGCATTGCTGCTAAAGAAGAAGGGGTATCTTTGGATAAAAGAATGTCTAAAGGTGAAGAATTATTCACTGGTGTTGTCCCAATTTTGGTTGAATTAGATGGTGATGTTAATGGTCACAAATTTTCTGTCTCCGGTGAAGGTGAAGGTGATGCTACTTACGGTAAATTGACCTTAAAATTTATTTGTACTACTGGTAAATTGCCAGTTCCATGGCCAACCTTAGTCACTACTTTCGGTTATGGTGTTCAATGTTTTGCTAGATACCCAGATCATATGAAACAACATGACTTTTTCAAGTCTGCCATGCCAGAAGGTTATGTTCAAGAAAGAACTATTTTTTTCAAAGATGACGGTAACTACAAGACCAGAGCTGAAGTCAAGTTTGAAGGTGATACCTTAGTTAATAGAATCGAATTAAAAGGTATTGATTTTAAAGAAGATGGTAACATTTTAGGTCACAAATTGGAATACAACTATAACTCTCACAATGTTTACATCATGGCTGACAAACAAAAGAATGGTATCAAAGTTAACTTCAAAATTAGACACAACATTGAAGATGGTTCTGTTCAATTAGCTGACCATTATCAACAAAATACTCCAATTGGTGATGGTCCAGTCTTGTTACCAGACAACCATTACTTATCCACTCAATCTGCCTTATCCAAAGATCCAAACGAAAAGAGAGACCACATGGTCTTGTTAGAATTTGTTACTGCTGCTGGTATTACCCATGGTATGGATGAATTGTACAAAGAGCAAAAGCTCATTTCTGAAGAGGACTTGATGAAATTATCAACTGTCCTATTATCTGCCGGTTTAGCCTGGACTACTTTGGCCCAATTTTCCAACAGTACATCTGCTTCTTCCACCGATGTCACTTCCTCCTCTTCCATCTCCACTTCCTCTGGCTCAGTAACTATCACATCTTCTGAAGCTCCAGAATCCGACAACGGTACCAGCACAGCTGCACCAACTGAAACCTCAACAGAGGCTCCAACCACTGCTATCCCAACTAACGGTACCTCTACTGAAGCTCCAACTGATACTACTTCTGAAGCTCCAACCACTGCTATCCCAACTAACGGTACCTCTACTGAAGCTCCAACTGATACTACTACTGAAGCTCCAACCACCGCTCTTCCAACTAACGGTACTTCTACTGAAGCTCCAACTGATACTACTACTGAAGCTCCAACCACCGGTCTTCCAACCAACGGTACCACTTCAGCTTTCCCACCAACTACATCTTTGCCACCAAGCAACACAACCACCACTCCTCCTTACAACCCATCTACTGACTACACCACTGACTACACCGTAGTCACTGAATATACTACTTACTGTCCAGAACCAACCACTTTCACCACAAACGGTAAGACTTACACTGTCACTGAACCAACCACATTGACTATCACTGACTGTCCATGCACCATTGAGAAGCCAACAACCACATCAACCACCGAATACACTGTAGTCACTGAGTACACTACTTACTGTCCAGAACCAACCACTTTCACCACAAACGGTAAGACTTACACTGTCACTGAACCAACCACTTTGACTATCACTGACTGTCCATGCACCATTGAGAAGCCAACAACCACATCAACCACCGAATACACTGTAGTCACTGAGTACACTACTTACTGTCCAGAACCAACCACTTTCACCACAAACGGTAAGACTTACACTGTCACTGAACCAACCACTTTGACTATCACTGACTGTCCATGTACTATTGAAAAGAGCGAAGCCCCTGAGTCTTCTGTCCCAGTTACCGAATCTAAGGGCACTACCACCAAAGAAACAGGTGTTACTACCAAACAAACCACAGCCAACCCAAGTCTAACTGTCTCCACAGTCGTCCCAGTTTCATCCTCTGCTTCTTCTCATTCCGTTGTCATCAACAGTAACGGTGCTAACGTCGTCGTTCCAGGTGCTTTAGGTTTGGCTGGTGTTGCTATGTTATTCTTATAA |
| S2 | ATGAGATTTCCTTCAATTTTTACTGCAGTTTTATTCGCAGCATCCTCCGCATTAGCTGCTCCAGTCAACACTACAACAGAAGATGAAACGGCACAAATTCCGGCTGAAGCTGTCATCGGTTACTTAGATTTAGAAGGGGATTTCGATGTTGCTGTTTTGCCATTTTCCAACAGCACAAATAACGGGTTATTGTTTATAAATACTACTATTGCCAGCATTGCTGCTAAAGAAGAAGGGGTATCTTTGGATAAAAGATACCCATATGATGTCCCCGACTATGCCATGTCTAAAGGTGAAGAATTATTCACTGGTGTTGTCCCAATTTTGGTTGAATTAGATGGTGATGTTAATGGTCACAAATTTTCTGTCTCCGGTGAAGGTGAAGGTGATGCTACTTACGGTAAATTGACCTTAAAATTTATTTGTACTACTGGTAAATTGCCAGTTCCATGGCCAACCTTAGTCACTACTTTCGGTTATGGTGTTCAATGTTTTGCTAGATACCCAGATCATATGAAACAACATGACTTTTTCAAGTCTGCCATGCCAGAAGGTTATGTTCAAGAAAGAACTATTTTTTTCAAAGATGACGGTAACTACAAGACCAGAGCTGAAGTCAAGTTTGAAGGTGATACCTTAGTTAATAGAATCGAATTAAAAGGTATTGATTTTAAAGAAGATGGTAACATTTTAGGTCACAAATTGGAATACAACTATAACTCTCACAATGTTTACATCATGGCTGACAAACAAAAGAATGGTATCAAAGTTAACTTCAAAATTAGACACAACATTGAAGATGGTTCTGTTCAATTAGCTGACCATTATCAACAAAATACTCCAATTGGTGATGGTCCAGTCTTGTTACCAGACAACCATTACTTATCCACTCAATCTGCCTTATCCAAAGATCCAAACGAAAAGAGAGACCACATGGTCTTGTTAGAATTTGTTACTGCTGCTGGTATTACCCATGGTATGGATGAATTGTACAAAGAGCAAAAGCTCATTTCTGAAGAGGACTTGGGTGGAGGAGGCTCTGGTGGAGGCGGTAGCGGAGGCGGAGGGTCGTTTTCCAACAGTACATCTGCTTCTTCCACCGATGTCACTTCCTCCTCTTCCATCTCCACTTCCTCTGGCTCAGTAACTATCACATCTTCTGAAGCTCCAGAATCCGACAACGGTACCAGCACAGCTGCACCAACTGAAACCTCAACAGAGGCTCCAACCACTGCTATCCCAACTAACGGTACCTCTACTGAAGCTCCAACTGATACTACTTCTGAAGCTCCAACCACTGCTATCCCAACTAACGGTACCTCTACTGAAGCTCCAACTGATACTACTACTGAAGCTCCAACCACCGCTCTTCCAACTAACGGTACTTCTACTGAAGCTCCAACTGATACTACTACTGAAGCTCCAACCACCGGTCTTCCAACCAACGGTACCACTTCAGCTTTCCCACCAACTACATCTTTGCCACCAAGCAACACAACCACCACTCCTCCTTACAACCCATCTACTGACTACACCACTGACTACACCGTAGTCACTGAATATACTACTTACTGTCCAGAACCAACCACTTTCACCACAAACGGTAAGACTTACACTGTCACTGAACCAACCACATTGACTATCACTGACTGTCCATGCACCATTGAGAAGCCAACAACCACATCAACCACCGAATACACTGTAGTCACTGAGTACACTACTTACTGTCCAGAACCAACCACTTTCACCACAAACGGTAAGACTTACACTGTCACTGAACCAACCACTTTGACTATCACTGACTGTCCATGCACCATTGAGAAGCCAACAACCACATCAACCACCGAATACACTGTAGTCACTGAGTACACTACTTACTGTCCAGAACCAACCACTTTCACCACAAACGGTAAGACTTACACTGTCACTGAACCAACCACTTTGACTATCACTGACTGTCCATGTACTATTGAAAAGAGCGAAGCCCCTGAGTCTTCTGTCCCAGTTACCGAATCTAAGGGCACTACCACCAAAGAAACAGGTGTTACTACCAAACAAACCACAGCCAACCCAAGTCTAACTGTCTCCACAGTCGTCCCAGTTTCATCCTCTGCTTCTTCTCATTCCGTTGTCATCAACAGTAACGGTGCTAACGTCGTCGTTCCAGGTGCTTTAGGTTTGGCTGGTGTTGCTATGTTATTCTTATAA |
| S3 | ATGAAATTATCAACTGTCCTATTATCTGCCGGTTTAGCCTGGACTACTTTGGCCCAATCTAAAGGTGAAGAATTATTCACTGGTGTTGTCCCAATTTTGGTTGAATTAGATGGTGATGTTAATGGTCACAAATTTTCTGTCTCCGGTGAAGGTGAAGGTGATGCTACTTACGGTAAATTGACCTTAAAATTTATTTGTACTACTGGTAAATTGCCAGTTCCATGGCCAACCTTAGTCACTACTTTCGGTTATGGTGTTCAATGTTTTGCTAGATACCCAGATCATATGAAACAACATGACTTTTTCAAGTCTGCCATGCCAGAAGGTTATGTTCAAGAAAGAACTATTTTTTTCAAAGATGACGGTAACTACAAGACCAGAGCTGAAGTCAAGTTTGAAGGTGATACCTTAGTTAATAGAATCGAATTAAAAGGTATTGATTTTAAAGAAGATGGTAACATTTTAGGTCACAAATTGGAATACAACTATAACTCTCACAATGTTTACATCATGGCTGACAAACAAAAGAATGGTATCAAAGTTAACTTCAAAATTAGACACAACATTGAAGATGGTTCTGTTCAATTAGCTGACCATTATCAACAAAATACTCCAATTGGTGATGGTCCAGTCTTGTTACCAGACAACCATTACTTATCCACTCAATCTGCCTTATCCAAAGATCCAAACGAAAAGAGAGACCACATGGTCTTGTTAGAATTTGTTACTGCTGCTGGTATTACCCATGGTATGGATGAATTGTACAAAGAGCAAAAGCTCATTTCTGAAGAGGACTTGGGTGGAGGAGGCTCTGGTGGAGGCGGTAGCGGAGGCGGAGGGTTTTCCAACAGTACATCTGCTTCTTCCACCGATGTCACTTCCTCCTCTTCCATCTCCACTTCCTCTGGCTCAGTAACTATCACATCTTCTGAAGCTCCAGAATCCGACAACGGTACCAGCACAGCTGCACCAACTGAAACCTCAACAGAGGCTCCAACCACTGCTATCCCAACTAACGGTACCTCTACTGAAGCTCCAACTGATACTACTTCTGAAGCTCCAACCACTGCTATCCCAACTAACGGTACCTCTACTGAAGCTCCAACTGATACTACTACTGAAGCTCCAACCACCGCTCTTCCAACTAACGGTACTTCTACTGAAGCTCCAACTGATACTACTACTGAAGCTCCAACCACCGGTCTTCCAACCAACGGTACCACTTCAGCTTTCCCACCAACTACATCTTTGCCACCAAGCAACACAACCACCACTCCTCCTTACAACCCATCTACTGACTACACCACTGACTACACCGTAGTCACTGAATATACTACTTACTGTCCAGAACCAACCACTTTCACCACAAACGGTAAGACTTACACTGTCACTGAACCAACCACATTGACTATCACTGACTGTCCATGCACCATTGAGAAGCCAACAACCACATCAACCACCGAATACACTGTAGTCACTGAGTACACTACTTACTGTCCAGAACCAACCACTTTCACCACAAACGGTAAGACTTACACTGTCACTGAACCAACCACTTTGACTATCACTGACTGTCCATGCACCATTGAGAAGCCAACAACCACATCAACCACCGAATACACTGTAGTCACTGAGTACACTACTTACTGTCCAGAACCAACCACTTTCACCACAAACGGTAAGACTTACACTGTCACTGAACCAACCACTTTGACTATCACTGACTGTCCATGTACTATTGAAAAGAGCGAAGCCCCTGAGTCTTCTGTCCCAGTTACCGAATCTAAGGGCACTACCACCAAAGAAACAGGTGTTACTACCAAACAAACCACAGCCAACCCAAGTCTAACTGTCTCCACAGTCGTCCCAGTTTCATCCTCTGCTTCTTCTCATTCCGTTGTCATCAACAGTAACGGTGCTAACGTCGTCGTTCCAGGTGCTTTAGGTTTGGCTGGTGTTGCTATGTTATTCTTATAA |
| S4 | ATGAGATTTCCTTCAATTTTTACTGCAGTTTTATTCGCAGCATCCTCCGCATTAGCTGCTCCAGTCAACACTACAACAGAAGATGAAACGGCACAAATTCCGGCTGAAGCTGTCATCGGTTACTTAGATTTAGAAGGGGATTTCGATGTTGCTGTTTTGCCATTTTCCAACAGCACAAATAACGGGTTATTGTTTATAAATACTACTATTGCCAGCATTGCTGCTAAAGAAGAAGGGGTATCTTTGGATAAAAGATACCCATATGATGTCCCCGACTATGCCTCTAAAGGTGAAGAATTATTCACTGGTGTTGTCCCAATTTTGGTTGAATTAGATGGTGATGTTAATGGTCACAAATTTTCTGTCTCCGGTGAAGGTGAAGGTGATGCTACTTACGGTAAATTGACCTTAAAATTTATTTGTACTACTGGTAAATTGCCAGTTCCATGGCCAACCTTAGTCACTACTTTCGGTTATGGTGTTCAATGTTTTGCTAGATACCCAGATCATATGAAACAACATGACTTTTTCAAGTCTGCCATGCCAGAAGGTTATGTTCAAGAAAGAACTATTTTTTTCAAAGATGACGGTAACTACAAGACCAGAGCTGAAGTCAAGTTTGAAGGTGATACCTTAGTTAATAGAATCGAATTAAAAGGTATTGATTTTAAAGAAGATGGTAACATTTTAGGTCACAAATTGGAATACAACTATAACTCTCACAATGTTTACATCATGGCTGACAAACAAAAGAATGGTATCAAAGTTAACTTCAAAATTAGACACAACATTGAAGATGGTTCTGTTCAATTAGCTGACCATTATCAACAAAATACTCCAATTGGTGATGGTCCAGTCTTGTTACCAGACAACCATTACTTATCCACTCAATCTGCCTTATCCAAAGATCCAAACGAAAAGAGAGACCACATGGTCTTGTTAGAATTTGTTACTGCTGCTGGTATTACCCATGGTATGGATGAATTGTACAAAGAGCAAAAGCTCATTTCTGAAGAGGACTTGGGTGGAGGAGGCTCTGGTGGAGGCGGTAGCGGAGGCGGAGGGTCGGCTCTTCCAACTAACGGTACTTCTACTGAAGCTCCAACTGATACTACTACTGAAGCTCCAACCACCGGTCTTCCAACCAACGGTACCACTTCAGCTTTCCCACCAACTACATCTTTGCCACCAAGCAACACAACCACCACTCCTCCTTACAACCCATCTACTGACTACACCACTGACTACACCGTAGTCACTGAATATACTACTTACTGTCCAGAACCAACCACTTTCACCACAAACGGTAAGACTTACACTGTCACTGAACCAACCACATTGACTATCACTGACTGTCCATGCACCATTGAGAAGCCAACAACCACATCAACCACCGAATACACTGTAGTCACTGAGTACACTACTTACTGTCCAGAACCAACCACTTTCACCACAAACGGTAAGACTTACACTGTCACTGAACCAACCACTTTGACTATCACTGACTGTCCATGCACCATTGAGAAGCCAACAACCACATCAACCACCGAATACACTGTAGTCACTGAGTACACTACTTACTGTCCAGAACCAACCACTTTCACCACAAACGGTAAGACTTACACTGTCACTGAACCAACCACTTTGACTATCACTGACTGTCCATGTACTATTGAAAAGAGCGAAGCCCCTGAGTCTTCTGTCCCAGTTACCGAATCTAAGGGCACTACCACCAAAGAAACAGGTGTTACTACCAAACAAACCACAGCCAACCCAAGTCTAACTGTCTCCACAGTCGTCCCAGTTTCATCCTCTGCTTCTTCTCATTCCGTTGTCATCAACAGTAACGGTGCTAACGTCGTCGTTCCAGGTGCTTTAGGTTTGGCTGGTGTTGCTATGTTATTCTTATAA |
| S5 | ATGAAATTATCAACTGTCCTATTATCTGCCGGTTTAGCCTGGACTACTTTGGCCCAATCTAAAGGTGAAGAATTATTCACTGGTGTTGTCCCAATTTTGGTTGAATTAGATGGTGATGTTAATGGTCACAAATTTTCTGTCTCCGGTGAAGGTGAAGGTGATGCTACTTACGGTAAATTGACCTTAAAATTTATTTGTACTACTGGTAAATTGCCAGTTCCATGGCCAACCTTAGTCACTACTTTCGGTTATGGTGTTCAATGTTTTGCTAGATACCCAGATCATATGAAACAACATGACTTTTTCAAGTCTGCCATGCCAGAAGGTTATGTTCAAGAAAGAACTATTTTTTTCAAAGATGACGGTAACTACAAGACCAGAGCTGAAGTCAAGTTTGAAGGTGATACCTTAGTTAATAGAATCGAATTAAAAGGTATTGATTTTAAAGAAGATGGTAACATTTTAGGTCACAAATTGGAATACAACTATAACTCTCACAATGTTTACATCATGGCTGACAAACAAAAGAATGGTATCAAAGTTAACTTCAAAATTAGACACAACATTGAAGATGGTTCTGTTCAATTAGCTGACCATTATCAACAAAATACTCCAATTGGTGATGGTCCAGTCTTGTTACCAGACAACCATTACTTATCCACTCAATCTGCCTTATCCAAAGATCCAAACGAAAAGAGAGACCACATGGTCTTGTTAGAATTTGTTACTGCTGCTGGTATTACCCATGGTATGGATGAATTGTACAAAGAGCAAAAGCTCATTTCTGAAGAGGACTTGGGTGGAGGAGGCTCTGGTGGAGGCGGTAGCGGAGGCGGAGGGTCGGCTCTTCCAACTAACGGTACTTCTACTGAAGCTCCAACTGATACTACTACTGAAGCTCCAACCACCGGTCTTCCAACCAACGGTACCACTTCAGCTTTCCCACCAACTACATCTTTGCCACCAAGCAACACAACCACCACTCCTCCTTACAACCCATCTACTGACTACACCACTGACTACACCGTAGTCACTGAATATACTACTTACTGTCCAGAACCAACCACTTTCACCACAAACGGTAAGACTTACACTGTCACTGAACCAACCACATTGACTATCACTGACTGTCCATGCACCATTGAGAAGCCAACAACCACATCAACCACCGAATACACTGTAGTCACTGAGTACACTACTTACTGTCCAGAACCAACCACTTTCACCACAAACGGTAAGACTTACACTGTCACTGAACCAACCACTTTGACTATCACTGACTGTCCATGCACCATTGAGAAGCCAACAACCACATCAACCACCGAATACACTGTAGTCACTGAGTACACTACTTACTGTCCAGAACCAACCACTTTCACCACAAACGGTAAGACTTACACTGTCACTGAACCAACCACTTTGACTATCACTGACTGTCCATGTACTATTGAAAAGAGCGAAGCCCCTGAGTCTTCTGTCCCAGTTACCGAATCTAAGGGCACTACCACCAAAGAAACAGGTGTTACTACCAAACAAACCACAGCCAACCCAAGTCTAACTGTCTCCACAGTCGTCCCAGTTTCATCCTCTGCTTCTTCTCATTCCGTTGTCATCAACAGTAACGGTGCTAACGTCGTCGTTCCAGGTGCTTTAGGTTTGGCTGGTGTTGCTATGTTATTCTTATAA |
| aaBGL | ATGAGATTTCCTTCAATTTTTACTGCAGTTTTATTCGCAGCATCCTCCGCATTAGCTGCTCCAGTCAACACTACAACAGAAGATGAAACGGCACAAATTCCGGCTGAAGCTGTCATCGGTTACTTAGATTTAGAAGGGGATTTCGATGTTGCTGTTTTGCCATTTTCCAACAGCACAAATAACGGGTTATTGTTTATAAATACTACTATTGCCAGCATTGCTGCTAAAGAAGAAGGGGTATCTTTGGATAAAAGAGATGTCGACGAATTCGATGAACTGGCGTTCTCTCCTCCTTTCTACCCCTCTCCGTGGGCCAATGGCCAGGGAGAGTGGGCGGAAGCCTACCAGCGTGCAGTGGCCATTGTATCCCAGATGACTCTGGATGAGAAGGTCAACCTGACCACCGGAACTGGATGGGAGCTGGAGAAGTGCGTCGGTCAGACTGGTGGTGTCCCAAGACTGAACATCGGTGGCATGTGTCTTCAGGACAGTCCCTTGGGAATTCGTGATAGTGACTACAATTCGGCTTTCCCTGCTGGTGTCAACGTTGCTGCGACATGGGACAAGAACCTTGCTTATCTACGTGGTCAGGCTATGGGTCAAGAGTTCAGTGACAAAGGAATTGATGTTCAATTGGGACCGGCCGCGGGTCCCCTCGGCAGGAGCCCTGATGGAGGTCGCAACTGGGAAGGTTTCTCTCCAGACCCGGCTCTTACTGGTGTGCTCTTTGCGGAAACTATTAAGGGTATTCAAGACGCTGGTGTCGTGGCGACAGCCAAGCATTACATTCTCAATGAGCAAGAGCATTTCCGCCAGGTCGCAGAGGCTGCGGGCTACGGATTCAATATCTCCGACACGATCAGCTCTAACGTTGATGACAAGACCATTCATGAAATGTACCTCTGGCCCTTCGCGGATGCCGTTCGCGCCGGCGTTGGCGCCATCATGTGTTCCTACAACCAGATCAACAACAGCTACGGTTGCCAGAACAGTTACACTCTGAACAAGCTTCTGAAGGCCGAGCTCGGCTTCCAGGGCTTTGTGATGTCTGACTGGGGTGCTCACCACAGTGGTGTTGGCTCTGCTTTGGCCGGCTTGGATATGTCAATGCCTGGCGATATCACCTTCGATTCTGCCACTAGTTTCTGGGGTACCAACCTGACCATTGCTGTGCTCAACGGTACCGTCCCGCAGTGGCGCGTTGACGACATGGCTGTCCGTATCATGGCTGCCTACTACAAGGTTGGCCGCGACCGCCTGTACCAGCCGCCTAACTTCAGCTCCTGGACTCGCGATGAATACGGCTTCAAGTATTTCTACCCCCAGGAAGGGCCCTATGAGAAGGTCAATCACTTTGTCAATGTGCAGCGCAACCACAGCGAGGTTATTCGCAAGTTGGGAGCAGACAGTACTGTTCTACTGAAGAACAACAATGCCCTGCCGCTGACCGGAAAGGAGCGCAAAGTTGCGATCCTGGGTGAAGATGCTGGATCCAACTCGTACGGTGCCAATGGCTGCTCTGACCGTGGCTGTGACAACGGTACTCTTGCTATGGCTTGGGGTAGCGGCACTGCCGAATTCCCATATCTCGTGACCCCTGAGCAGGCTATTCAAGCCGAGGTGCTCAAGCATAAGGGCAGCGTCTACGCCATCACGGACAACTGGGCGCTGAGCCAGGTGGAGACCCTCGCTAAACAAGCCAGTGTCTCTCTTGTATTTGTCAACTCGGACGCGGGAGAGGGCTATATCTCCGTGGACGGAAACGAGGGCGACCGCAACAACCTCACCCTCTGGAAGAACGGCGACAACCTCATCAAGGCTGCTGCAAACAACTGCAACAACACCATCGTTGTCATCCACTCCGTTGGACCTGTTTTGGTTGACGAGTGGTATGACCACCCCAACGTTACTGCCATCCTCTGGGCGGGCTTGCCTGGCCAGGAGTCTGGCAACTCCTTGGCTGACGTGCTCTACGGCCGCGTCAACCCGGGCGCCAAATCTCCATTCACCTGGGGCAAGACGAGGGAGGCGTACGGGGATTACCTTGTCCGTGAGCTCAACAACGGCAACGGAGCTCCCCAAGATGATTTCTCGGAAGGTGTTTTCATTGACTACCGCGGATTCGACAAGCGCAATGAGACCCCGATCTACGAGTTCGGACATGGTCTGAGCTACACCACTTTCAACTACTCTGGCCTTCACATCCAGGTTCTCAACGCTTCCTCCAACGCTCAAGTAGCCACTGAGACTGGCGCCGCTCCCACCTTCGGACAAGTCGGCAATGCCTCTGACTACGTGTACCCTGAGGGATTGACCAGAATCAGCAAGTTCATCTATCCCTGGCTTAATTCCACAGACCTGAAGGCCTCATCTGGCGACCCGTACTATGGAGTCGACACCGCGGAGCACGTGCCCGAGGGTGCTACTGATGGCTCTCCGCAGCCCGTTCTGCCTGCCGGTGGTGGCTCTGGTGGTAACCCGCGCCTCTACGATGAGTTGATCCGTGTTTCGGTGACAGTCAAGAACACTGGTCGTGTTGCCGGTGATGCTGTGCCTCAATTGTATGTTTCCCTTGGTGGACCCAATGAGCCCAAGGTTGTGTTGCGCAAATTCGACCGCCTCACCCTCAAGCCCTCCGAGGAAACGGTGTGGACGACTACCCTGACCCGCCGCGATCTGTCTAACTGGGACGTTGCGGCTCAGGACTGGGTCATCACTTCTTACCCGAAGAAGGTCCATGTTGGTAGCTCTTCGCGTCAGCTGCCCCTTCACGCGGCGCTCCCGAAGGTGCAATCTGGAGGTGGCGGTTCCGGAGGCGGAGGTTCAGGAGGCGGAGGTTCTGCACAGGAACTGACAACTATATGCGAGCAAATCCCCTCACCAACTTTAGAATCGACGCCGTACTCTTTGTCAACGACTACTATTTTGGCCAACGGGAAGGCAATGCAAGGAGTTTTTGAATATTACAAATCAGTAACGTTTGTCAGTAATTGCGGTTCTCACCCCTCAACAACTAGCAAAGGCAGCCCCATAAACACACAGTATGTTTTTAAGGACAATAGCTCGACGATTGAAGGTAGATACCCATACGACGTTCCAGACTACGCTCTGCAGGCTAGTGGTGGAGGAGGCTCTGGTGGAGGCGGTAGCGGAGGCGGAGGGTCGTCTAAAGGTGAAGAATTATTCACTGGTGTTGTCCCAATTTTGGTTGAATTAGATGGTGATGTTAATGGTCACAAATTTTCTGTCTCCGGTGAAGGTGAAGGTGATGCTACTTACGGTAAATTGACCTTAAAATTTATTTGTACTACTGGTAAATTGCCAGTTCCATGGCCAACCTTAGTCACTACTTTCGGTTATGGTGTTCAATGTTTTGCTAGATACCCAGATCATATGAAACAACATGACTTTTTCAAGTCTGCCATGCCAGAAGGTTATGTTCAAGAAAGAACTATTTTTTTCAAAGATGACGGTAACTACAAGACCAGAGCTGAAGTCAAGTTTGAAGGTGATACCTTAGTTAATAGAATCGAATTAAAAGGTATTGATTTTAAAGAAGATGGTAACATTTTAGGTCACAAATTGGAATACAACTATAACTCTCACAATGTTTACATCATGGCTGACAAACAAAAGAATGGTATCAAAGTTAACTTCAAAATTAGACACAACATTGAAGATGGTTCTGTTCAATTAGCTGACCATTATCAACAAAATACTCCAATTGGTGATGGTCCAGTCTTGTTACCAGACAACCATTACTTATCCACTCAATCTGCCTTATCCAAAGATCCAAACGAAAAGAGAGACCACATGGTCTTGTTAGAATTTGTTACTGCTGCTGGTATTACCCATGGTATGGATGAATTGTACAAA |
| VB221020-1396kuv | <https://en.vectorbuilder.com/vector/VB221020-1396kuv.html> |
| VB231023-1165htk | <https://en.vectorbuilder.com/vector/VB231023-1165htk.html> |

Table S2. Primers and oligonucleotides used for host modification

| Primers/dsDNA fragment | Sequence |
| --- | --- |
| *URA3* KO_gRNA spacer | CGTTCGAAACTTCTCCGCAGTGAAAGATAAATGATCGAGTAAAAAATTGTACTTGGGTTTTAGAGCTAGAAATAGCAAGTTAAAATAAGGCTAGTCCGTTATCAACTTGAAAAA |
| *URA3* KO_donor | TCTTAACCCAACTGCACAGAACAAAAACCTGCAGGAAACGAAGATAAATCAAAACTGTATTATAAGTAAATGCATGTATACTAAACTCACAAATTAGAGC |
| pRS42H_backbone amplification_F | GTTTTAGAGCTAGAAATAGCAAG |
| pRS42H_backbone amplification_R | GATCATTTATCTTTCACTGC |
| *AGA1* overexpression_gRNA spacer | TCGGCGTTCGAAACTTCTCCGCAGTGAAAGATAAATGATCAAATTCCGAAGCATGTAGGGGTTTTAGAGCTAGAAATAGCAAGTTAAAATAAGGCTAGTC |
| *AGA1* overexpression_donor amplification_F1 | TTGGTCGGAACGACCAAGCTTCAATTTATAAGTTTATCAT |
| *AGA1* overexpression_donor amplification_F2 | ACAATAGCTAATTTACGTTTCCCTACGGTATTGGTCGGA |
| *AGA1* overexpression_donor amplification_R1 | GGTAGGTAAAATGAGCGAAAGATAATGTCATTCGAAACCAAGTTC |
| *AGA1* overexpression_donor amplification_R2 | ATTAGTTAATCCCAACAATATTGTGAACAGGTAGGTAAAATGAG |
| *AGA1* overexpression_confirm_F | CTTAGCGCAACTACAGAGAA |
| *AGA1* overexpression_confirm_R | CCGTTTCATGCATACTGG |
| *CCW12* KO_gRNA spacer | TCGGCGTTCGAAACTTCTCCGCAGTGAAAGATAAATGATCTAAGAGGTGACAGAGTGAGTGTTTTAGAGCTAGAAATAGCAAGTTAAAATAAGGCTAGTC |
| *CCW12* KO_donor | TTGGCTAAGAGGCACTGCGTATACTTCAAGGTACCCCTGTGTTTTGAAAAAGGAGAATAAGAATTATTAGAACAATGTAGGATGGAAAGAAAGATTATCC |
| *CCW12* KO_confirm_F | AACCGGTCCTGACGTCACTGAAAAGATTTC |
| *CCW12* KO_confirm_R | CTTCCTTCCCTTCGAAGATCCTCCCG |
| *CCW14* KO_gRNA spacer | TCGGCGTTCGAAACTTCTCCGCAGTGAAAGATAAATGATCGTCTAGACATTTCTTGACGGGTTTTAGAGCTAGAAATAGCAAGTTAAAATAAGGCTAGTC |
| *CCW14* KO_donor | GCATTGGACCCATGCACCACATGCATTAGGACCCATTATTTCTTTTTTACATGGGTTAAGGTATCTATCTTTTATATTTCTTATTTCCTCTTCTTATATG |
| *CCW14* KO_confirm_F | AGGCGCCAATATTGACGTCTAGC |
| *CCW14* KO_confirm_R | AGACCGGTATCATCAGTCACATCAACA |
| *FYV5* KO_gRNA spacer | TCGGCGTTCGAAACTTCTCCGCAGTGAAAGATAAATGATCGTAAATATATACATATAGAGGTTTTAGAGCTAGAAATAGCAAGTTAAAATAAGGCTAGTC |
| *FYV5* KO_donor | ATCCAGCGCTGGCCTCAGATCCTCGAAGGTTTAGTCTTAAGCGACGATGCTTTATATATATTCAAAAAAAAAAAATAATAAAAATATGCAGTACCATTCCTAGTGATAGGCTGGCTATATGTATATATTTACGTAACTTTCACCACTATTCCCTATAAGGAAAAGCCGGATATCATTTCGATCTGCTTCAGCATGCTGTC |
| *FYV5* KO_confirm_F | CCATTCCTAGTGATAGGCTGG |
| *FYV5* KO_confirm_R | GCCAGTAGAGTTGGTGTCCTAGTAAG |

Table S3. Primers and oligonucleotides used for genome integration

| Primers/Spacer | Sequence |
| --- | --- |
| Genome integration_donor_F | TCCACTCCCCCATTTTTATCCGGATCTCTGAGTTTATCATTATCAATACTGCCATT |
| Genome integration_donor_R | GCGCGTTGGCCGATTCATTAATGCAGGGCCGCAGCTTGCAAATTAAA |
| Intergenic region _gRNA spacer | TGTGACAAAATAGAATCCAG |
| Genome integration confirm_F | AGGAGTGAGAATGTGCGTTAGC |
| Genome integration confirm_R | GGCAATGCAAATTCTTCCAGAC |

Table S4. Fermentation parameters of WT and LIP02

|  | WT | LIP02 |
| --- | --- | --- |
| Average OD_600_ at 24h | 7.79±0.40 | 9.07±0.04 |
| DCW at 24h (g/L) | 3.81±0.26 | 3.83±0.02 |
| Cellobiose consumed at 24h (g/L) | 15.54±0.59 | 22.74±2.34 |
| Specific degradation rate (g/g_cell_/h) | 0.17±0.00 | 0.28±0.02 |
